# Comparison of preprocessing techniques to reduce nontissue-related variations in hyperspectral reflectance imaging

**DOI:** 10.1117/1.JBO.27.10.106003

**Published:** 2022-10-07

**Authors:** Mark Witteveen, Henricus J. C. M. Sterenborg, Ton G. van Leeuwen, Maurice C. G. Aalders, Theo J. M. Ruers, Anouk L. Post

**Affiliations:** a the Netherlands Cancer Institute, Surgical Oncology, Amsterdam, The Netherlands; b University of Twente, Science and Technology, Nanobiophysics, Enschede, The Netherlands; c Amsterdam UMC, University of Amsterdam, Cancer Center Amsterdam, Amsterdam Cardiovascular Sciences, Department of Biomedical Engineering and Physics, Amsterdam, The Netherlands; d University of Amsterdam, Co van Ledden Hulsebosch Center, Amsterdam, The Netherlands

**Keywords:** hyperspectral, preprocessing, normalization, classification, scatter correction, cancer, glare, machine learning

## Abstract

**Significance:**

Hyperspectral reflectance imaging can be used in medicine to identify tissue types, such as tumor tissue. Tissue classification algorithms are developed based on, e.g., machine learning or principle component analysis. For the development of these algorithms, data are generally preprocessed to remove variability in data not related to the tissue itself since this will improve the performance of the classification algorithm. In hyperspectral imaging, the measured spectra are also influenced by reflections from the surface (glare) and height variations within and between tissue samples.

**Aim:**

To compare the ability of different preprocessing algorithms to decrease variations in spectra induced by glare and height differences while maintaining contrast based on differences in optical properties between tissue types.

**Approach:**

We compare eight preprocessing algorithms commonly used in medical hyperspectral imaging: standard normal variate, multiplicative scatter correction, min–max normalization, mean centering, area under the curve normalization, single wavelength normalization, first derivative, and second derivative. We investigate conservation of contrast stemming from differences in: blood volume fraction, presence of different absorbers, scatter amplitude, and scatter slope—while correcting for glare and height variations. We use a similarity metric, the overlap coefficient, to quantify contrast between spectra. We also investigate the algorithms for clinical datasets from the colon and breast.

**Conclusions:**

Preprocessing reduces the overlap due to glare and distance variations. In general, the algorithms standard normal variate, min–max, area under the curve, and single wavelength normalization are the most suitable to preprocess data used to develop a classification algorithm for tissue classification. The type of contrast between tissue types determines which of these four algorithms is most suitable.

## 1 Introduction

Hyperspectral imaging can be used in medical applications to distinguish tissue types based on differences in their spectral signature.^1^^–^^5^ Currently, many researchers use a variety of methods to develop classification algorithms for hyperspectral imaging, such as machine learning, statistical analysis, or fitting algorithms, which identify tissue types based on spectral differences between tissue types. The quality of the data used by the classification algorithm influences the accuracy and robustness of the developed algorithm. Ideally, the spectra used to distinguish tissue types are only influenced by the tissue composition, but unfortunately these spectra are also influenced by other factors, such as surface reflections and sample thickness variations. Removing influences not related to tissue composition is expected to improve the accuracy of the developed algorithm itself, and it would also make the developed algorithm more generalizable, because it does not depend on the amount of surface reflections and sample thickness variations present in the dataset used to develop the algorithm. Thus, reducing these influences is a valuable step in the development of classification algorithms for hyperspectral imaging.

**Fig. 1:**
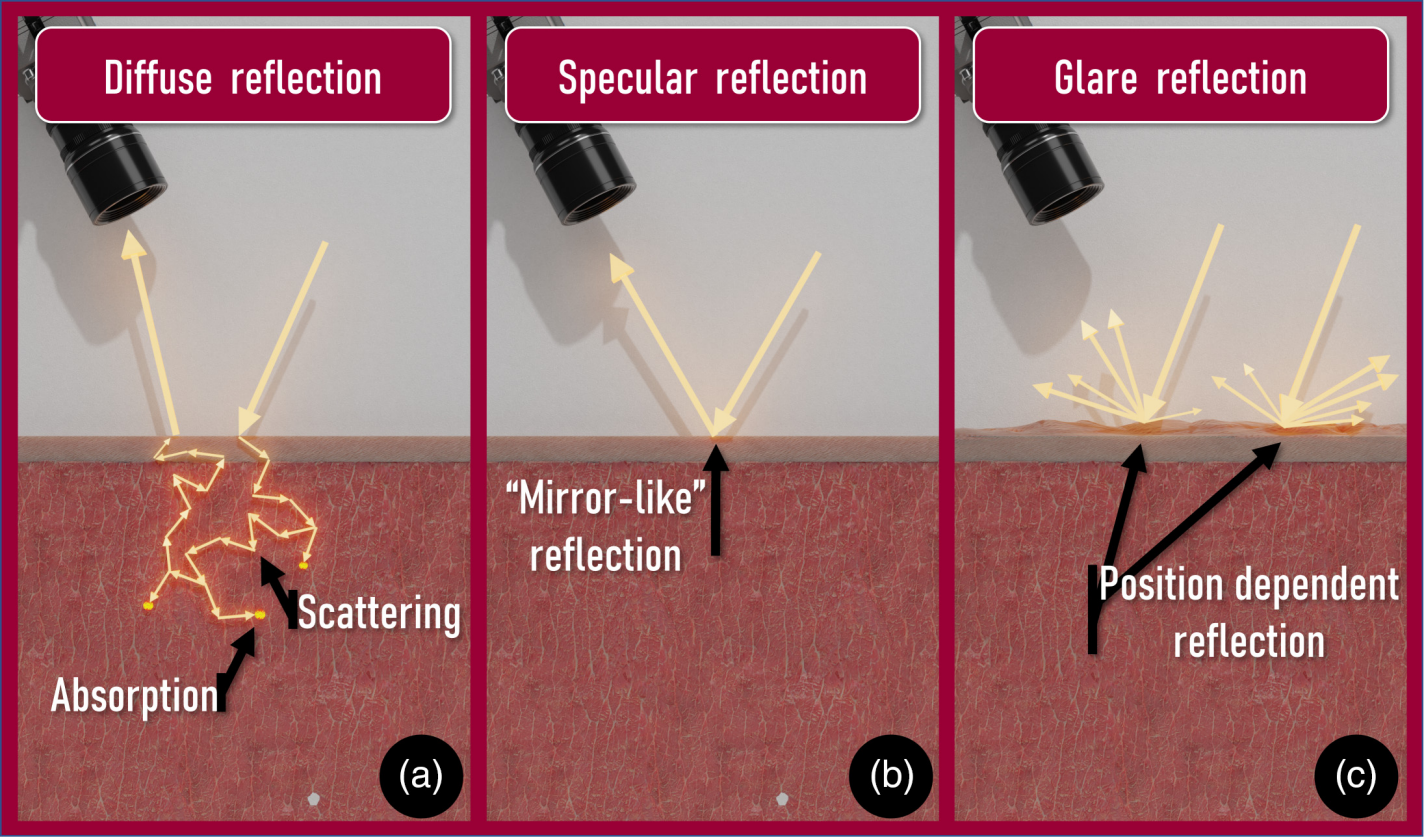
Three different types of reflected light: (a) diffuse reflectance, where the light travels through the tissue, (b) specular reflectance from a smooth tissue surface, and (c) glare as a result of surface roughness, where light is reflected from the surface in many directions. Only the light that reaches the sensor should be considered for the measured signal.

The measured reflectance is the sum of diffuse [Fig. 1(a)] and surface reflections [Figs. 1(b) and 1(c)]. Surface reflections can be divided into mirror-like specular reflections from a smooth tissue surface [Fig. 1(b)] and specular reflections from a rough tissue surface, known as glare [Fig. 1(c)]. Glare is a form of specular reflection, albeit on a much smaller scale, that is influenced by the roughness of the tissue surface. To prevent mirror-like specular reflections from a smooth surface, the light source and camera are generally placed at an angle with respect to each other. However, this will not prevent glare. Due to the surface roughness of the tissue, light from the tissue surface will be reflected in many directions of which some will be detected by the camera. Assuming surface roughness is not homogeneous, the orientation of the tissue sample with respect to the camera will influence the detected amount of glare. Thus, the amount of glare in a measured spectrum depends not only on the tissue composition but also on the geometry of the hyperspectral setup.

In *ex-vivo* settings with benchtop systems, the influence of surface reflections can be reduced by polarization filters and the influence of variable sample heights by surface profilometry. However, for *in-vivo* applications, data analysis should be fast to provide clinicians with real-time information. Surface profilometry takes additional time during a measurement and has to be redone frequently to allow the clinician to move the hyperspectral imaging device. Polarization filters not only remove glare but also reduce the intensity of the diffuse reflectance since the diffuse reflectance will be unpolarized. Thus, to obtain a good signal-to-noise ratio, this could increase measurement times, especially in low-light settings such as endoscopy. Instead of hardware solutions, the influence of surface reflections and sample height variations can also be reduced by preprocessing the measured spectra used by tissue classification algorithms.

In theory, when sophisticated algorithms such as convolutional neural networks are used on vast amounts of data, algorithms should be able to learn to identify tissue types in the presence of additional variations such as surface reflections. However, for clinical applications, algorithms are always developed with limited sample sizes, especially when hyperspectral imaging is used to identify tissue types since it requires correlation with histopathology, which is expensive and time-consuming. Algorithms developed on limited sample sizes with additional variations in the spectra that are not related to tissue composition could result in a poorer performance. Multiple studies have shown that, in general, preprocessing data before feeding it to a convolutional neural network improves the performance of the developed algorithm.^6^^–^^8^

**Fig. 2:**
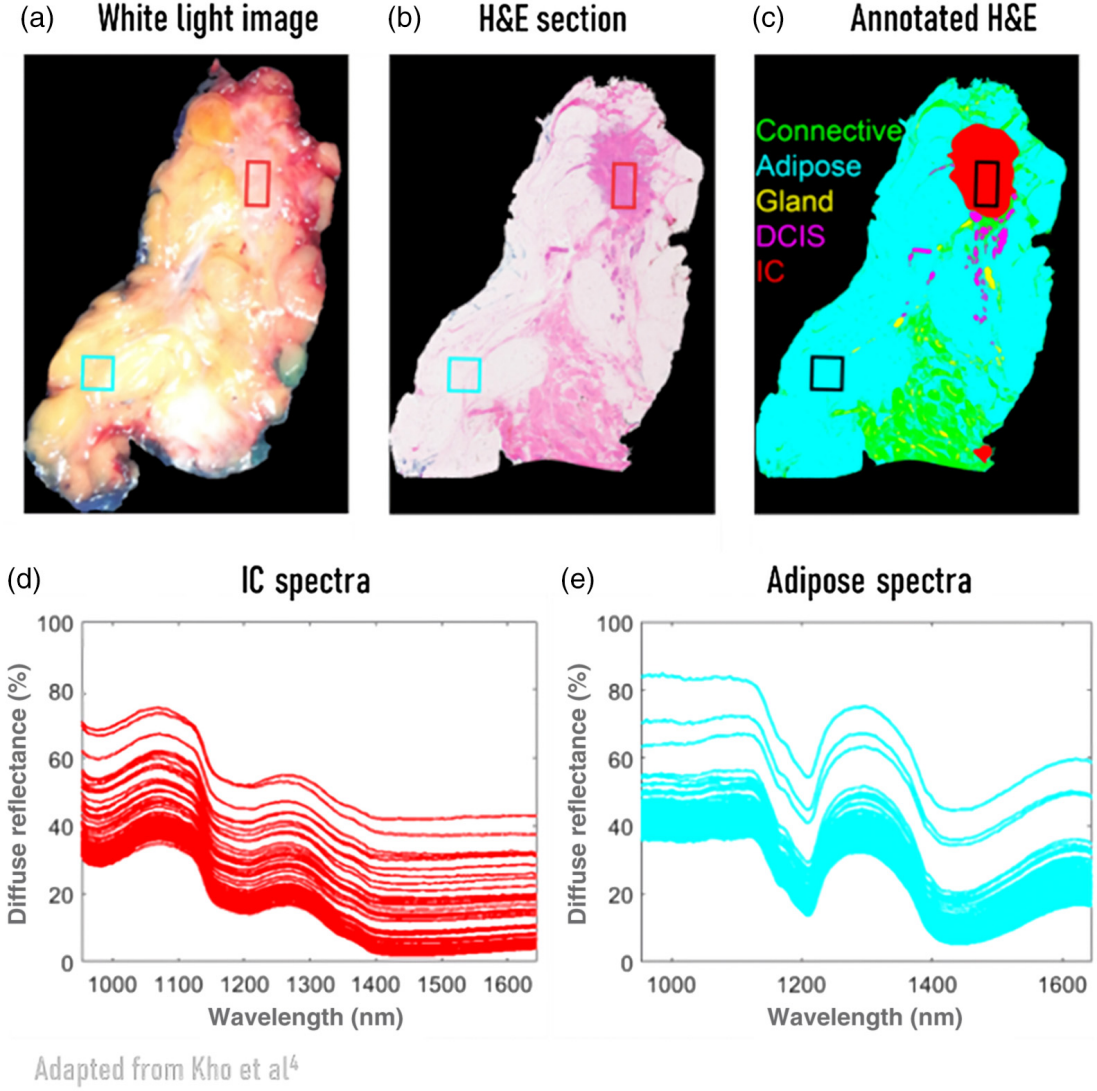
An illustration of variations in measured spectra, which are not directly related to tissue composition or sample thickness, obtained from resected breast tissue. (a) White light image of the tissue specimen, (b) stained H&E section, (c) image from the hyperspectral camera with annotations based on histopathology to indicate the tissue class of each pixel, (d) spectra from the red region of interest containing invasive carcinoma, (e) spectra from the blue region of interest containing adipose tissue. Because the size of these rectangles is small, no variation in tissue composition or height differences are expected. The variation between spectra within a rectangle are likely attributable to glare. Figure modified from Kho et al.^4^

An example of the influence of glare can be seen in the study by Kho et al.^4^ where they imaged breast tissue slices. In Fig. 2, spectra from two regions with different tissue types as indicated by the rectangles are shown. Within each rectangle, the tissue type is the same [Fig. 2(a)–2(c)], but large variations exist in the obtained spectra [Figs. 2(a) and 2(d)]. Because the size of these rectangles is small, no variation in tissue composition is expected. The large variations shown in Figs. 2(d) and 2(e) give the impression of a wavelength-independent offset. Specular reflection, and thus glare by extension, is related to the difference in refractive index of tissue and air. As the refractive index of tissue does not change dramatically with wavelength (for both pure water and pure fat it varies <2% over the entire 400 to 1600 nm range), the specular reflection and thus also the glare will only vary slightly with wavelength at lower wavelengths. Hence, glare would result in an offset, and thus the large variations in the measured spectra shown in Figs. 2(d) and 2(e) are likely to be attributed to glare.

The pipeline for classifying tissue using hyperspectral data can often be broken down into three steps: calibration, which is necessary to compensate for wavelength-dependent properties of the setup; preprocessing, which is needed to reduce unwanted variation in the data; and a classification algorithm, where the actual classification based on the data is made. For the development of a classification algorithm, there are several possibilities, such as fitting spectra with diffusion theory and using optical properties or principle component analysis. In recent years, machine learning is increasingly used in hyperspectral imaging to develop classification algorithms.

Hyperspectral imaging setups require calibration primarily to compensate for wavelength-dependent properties of the setup such as: sensor sensitivity, transmission of lenses used, variable properties of the light source, and the physical layout of the setup. Ideally, a calibration is done with a reference sample, performed at a location corresponding to the location of the imaged sample. However, in practice, this is rarely the case. Sample thickness variations introduce distance differences between the sample, light source, and camera. When the tissue is closer to the light source, the incident intensity will increase; if the tissue is closer to the camera, the surface area represented by a pixel on the camera will decrease. The character of these spectral variations is very different from the variations in glare: where glare adds an offset to the spectrum from within the sample, sample height variations influence the amount of light that is detected. Height differences will result in a multiplication of the entire spectrum with a wavelength-independent constant and introduce differences in the size of the tissue area that corresponds to a pixel in the camera.

Thus, glare and sample thickness variations introduce variations in the measured spectra, which are not related to tissue composition. This makes spectra from different tissue types harder to distinguish from each other. For the development of tissue classification algorithms in hyperspectral imaging, several preprocessing algorithms have been used to reduce the variations that are not related to tissue composition. An ideal preprocessing algorithm would reduce the differences in the spectra that are due to glare and height differences while retaining the spectral signatures that differ between tissue types. Currently, which preprocessing algorithm is more suitable for a specific clinical application is unknown. One approach of choosing a preprocessing algorithm would be to test combinations of preprocessing algorithms and classification algorithms and determine which combination results in the highest accuracy of the classifier. However, this approach (data-dredging—testing multiple hypotheses on a single dataset by extensively searching for the most optimal approach) could increase the probability of lucky shots and thereby nonreproducible results.^9^ Reducing the amount of considered preprocessing techniques and introducing selection criteria could reduce these effects. In this paper, we provide researchers with a solid basis to identify the most suitable preprocessing algorithm for their application.

By a literature search, we identified eight commonly used preprocessing algorithms applied to medical hyperspectral images: standard normal variate (SNV),^10^^–^^17^ multiplicative scatter correction (MSC),^18^^–^^20^ min-max normalization (MM),^16^^,^^21^^–^^29^ mean centering (MC),^30^^,^^31^ area under the curve normalization (AUC),^32^^–^^37^ single wavelength normalization (SW),^1^^,^^38^ first derivative (FD),^16^^,^^18^^,^^39^^–^^41^ and second derivative (SD).^42^^,^^43^ The details of each algorithm are discussed in Sec. 2.3.

In this study, synthetic reflection spectra (based on a simulated dataset resembling diffuse reflectance spectra from human tissue from 400 to 1600 nm) were created with known differences in optical properties from absorption [blood volume fraction (BVF) and presence of absorbers] and scattering (scatter amplitude and slope). For each set of tissue properties, 100 spectra with variable amounts of glare and variable height differences and noise were generated. Ideally, a preprocessing algorithm would reduce the differences in spectra within one tissue type while maintaining the differences in spectra between tissue types. To quantify how well an algorithm does this, we calculated the “overlap coefficient” of the spectra, a measure of similarity (ranging from 0 to 1), for any of combination of two sets of tissue properties. For two sets of spectra with different tissue properties, a reduced overlap coefficient after preprocessing would imply that variations due to glare and height differences are reduced, while the contrast related to the difference in optical properties is retained. Thus, a lower overlap coefficient would likely improve the discrimination ability of tissue classification algorithms. Finally, we investigate the effect of the preprocessing algorithms on clinical data, measured on colon^10^ and breast^11^ tissues to determine whether the trends we identify based on the synthetic data translate to clinical applications.

## 2 Methods

### 2.1 Synthetic Spectra Theoretical Background

The diffuse reflectance consists of light that has traveled though the tissue. For the illumination geometry commonly used in hyperspectral imaging, an infinite wide beam and infinite sample thickness can be assumed. Consequently, the diffuse reflectance ($R_{\text{diffuse}}(\lambda )$) of a homogeneous sample with $\mu_{a}\ll \mu_{s}^{\prime }$ can be approximated using diffusion theory as:^44^

$$R_{\text{diffuse}}(\lambda )=\frac{\alpha^{\prime }(\lambda )}{1+2k(1-\alpha^{\prime }(\lambda ))+(1+\frac{2k}{3})\sqrt{3(1-\alpha^{\prime }(\lambda ))}},$$ (1)

where k is the internal reflection coefficient due to the tissue–air refractive index mismatch and $\alpha^{\prime }(\lambda )$ is the transport albedo, which equals:

$$\alpha^{\prime }(\lambda )=\frac{\mu_{s}^{\prime }(\lambda )}{\mu_{s}^{\prime }(\lambda )+\mu_{a}(\lambda )}.$$ (2)

Here, $\mu_{s}^{\prime }$ is the reduced scattering coefficient and $\mu_{a}$ is the absorption coefficient, which both depend on the wavelength λ. The reduced scattering coefficient $\mu_{s}^{\prime }(\lambda )$ can be described as $\mu_{s}^{\prime }(\lambda )=a\cdot (\frac{\lambda }{\lambda_{0}}{)}^{-b}$, where a is the scatter amplitude, b is the scatter slope, and $\lambda_{0}$ is the reference wavelength to normalize the reduced scattering coefficient and make it dimensionless. In this paper, the reference wavelength was set to 500 nm.

For light incident perpendicular to a boundary where the refractive index changes from $n_{1}$ to $n_{2}$, the specular reflection $R_{s}$ is given by the Fresnel equation:

$$R_{s}(\lambda )={\left|\frac{n_{1}(\lambda )-n_{2}(\lambda )}{n_{1}(\lambda )+n_{2}(\lambda )}\right|}^{2}.$$ (3)

We assume that glare will be proportional to but smaller than $R_{s}$—depending on the surface roughness of the tissue, the tissue refractive index, and the illumination/detection geometry. We will simulate this effect as a fraction of $R_{s}$ reaching the detector, independent of the wavelength. For the synthetic spectra, glare is then simulated as

$$R_{\text{glare}}(\lambda )=R_{s}(\lambda )\cdot \text{rand}(0,1),$$ (4)

where $R_{s}$ is the maximum reflection given by Eq. (3), multiplied by a random number between 0 and 1 from a uniform distribution.

In most clinical applications, the imaged tissue specimen is not a flat surface. In addition to surface roughness, as discussed above, sample thickness variations introduce distance differences among the sample, light source, and camera. When the tissue is closer to the light source, the incident intensity per surface area will increase; if the tissue is closer to the camera, the surface area represented by a pixel on the camera will decrease. Thus, sample thickness variations have a multiplicative effect on the measured spectra. We model the light source as an isotropic point source, in which case the intensity decreases with the square of the distance from the point source ($d_{\text{source}}$). Glare can be seen as a form of specular (mirror-like) reflection and does not interact with the tissue, therefore the light can be modeled as having traveled the combined distance between the source and the tissue ($d_{\text{source}}$) and from the tissue to the detector ($d_{\det}$). Assuming the distance between the source and the tissue is equal to the distance between the tissue and the detector (d), the effect of distance on glare is then equal to the inverse square law over the combined total distance:

$$\beta_{\text{glare}}=\frac{1}{{\left(d_{\text{source}}+d_{\det}\right)}^{2}}=\frac{1}{4d^{2}}.$$ (5)

Diffuse light interacts with the tissue and can therefore not be modeled in the same manner. After the light has entered the tissue, under the diffusion approximation the light will exit the tissue with isotropic radiance.^45^ For isotropic radiance, the diffusely reflected light can be modeled using the inverse square law:

$$\beta_{\text{diffuse}}=\frac{1}{d_{\text{source}}^{2}\cdot d_{\det}^{2}}=\frac{1}{d^{4}}.$$ (6)

Thus, glare and distance scale with different factors. The total measured reflectance $R_{\mathrm{tot}}(\lambda )$ can be simulated as the sum of the diffusely reflected light ($R_{\text{diffuse}}(\lambda )$) multiplied by the scaling factor for the distance ($\beta_{\text{diffuse}}$), glare ($R_{\text{glare}}(\lambda )$) multiplied by the scaling factor for the distance ($\beta_{\text{glare}}$), and noise (γ):

$$R_{\mathrm{tot}}(\lambda )=[R_{\text{diffuse}}(\lambda )\cdot \beta_{\text{diffuse}}]+[R_{\text{glare}}(\lambda )\cdot \beta_{\text{glare}}]+\gamma (\lambda ).$$ (7)

### 2.2 Synthetic Spectra Generation

First, $R_{\text{diffuse}}$ was created from 400 to 1600 nm with a step size of 1 nm, using diffusion theory [Eq. (1)] and the absorption coefficients of water,^46^ fat,^47^ hemoglobin,^48^ and bilirubin^46^ in combination with the optical tissue parameters from Table 1. Sets of spectra were made in which the contrast between tissue types was either a varying BVF, the presence of different absorbers, a varying scatter amplitude (a), or a varying scatter slope (b). Table 1 specifies the properties used for each set of simulations. The values were chosen to simulate general soft tissue based on the review by Jacques.^49^

**Table 1:** Tissue parameters used to create the synthetic data, based on the review by Jacques.^49^ B, blood; F, fat; W, water; Bi, bilirubin; VF, volume fraction; *a*, scatter amplitude; *b*, scatter slope. For the oxygen saturation of blood, a value of 75% was used.

Contrast	Absorbers	Optical properties					
		BVF (%)	FVF (%)	WVF (%)	Bi (mg/dL)	a (${\mathrm{cm}}^{-1}$)	b
BVF	B + F + W	0.5; 2.0; 3.5; 5.0; 6.5	35	35	0	15.35	1.25
Presence of different absorbers	B	4.0	0	0	0	15.35	1.25
	B + W;		0	70	0		
	B + W + F;		35	35	0		
	B + W + Bi;		0	70	13.5		
	B + W + F + Bi		35	35	13.5		
Scatter amplitude	B + F + W	4.0	35	35	0	8; 12; 16; 20; 24	1.25
Scatter slope	B + F + W	4.0	35	35	0	15.35	0.500; 0.875; 1.250; 1.625; 2.00

Next, for each $R_{\text{diffuse}}$, 100 spectra with variable amounts of glare, sample heights, and noise were generated [Eq. (7)]. The specular reflection was calculated using the Fresnel equations for the reflection of light at an interface between two media with different optical properties—in this case water and air [Eq. (3)]. Glare was added as a variable fraction (between 0 and 1) of the specular reflection, using the “rand” function in MathLab (MathWorks, Natick, Massachusetts) picking values from a uniform distribution. The added glare is then defined in Eq. (4) as $R_{\text{glare}}$.

The simulated height differences were based on the height differences observed in the study of Kho et al.^11^ Within a single specimen, height differences of ∼1  cm were observed, and between specimens, height differences are between 1 and 5 cm. The detector in the benchtop system was placed at 30 cm from the measurement plate, giving a maximum distance variation of 20% between all the measurements. Thus, the maximum value for d in Eqs. (5) and (6) is equal to 1.2.

We simulated realistic white noise and added this to all the spectra to make our results more comparable to real-life measurements. From the clinical datasets of the breast^11^ and colon^10^ obtained previously within our group, a noise estimation was made. The noise estimation was done by taking the difference between a Savitzky–Golay smoothed reflectance spectrum and the unprocessed spectrum.^50^ This was done for the breast and colon datasets and then the average of those two was taken, which was approximated as

$${\text{Intensity}}_{\gamma }(\lambda )=1.0497\cdot 10^{-15}\cdot {\left|\lambda -\overset{¯}{\lambda }\right|}^{5}+9.5469\cdot 10^{-4},$$ (8)

$\overset{¯}{\lambda }$ is the mean wavelength, such that for a spectra from 400 to 1600 nm, $\overset{¯}{\lambda }=1000\;\mathrm{nm}$. The intensity of the noise is highest at the extremes of the spectra and reduces in the middle, as was seen in the clinical datasets. For the simulated spectra, we multiplied ${\text{Intensity}}_{\gamma }(\lambda )$ by a random number from a uniform distribution between −0.5 and 0.5 for each wavelength:

$$\gamma (\lambda )={\text{Intensity}}_{\gamma }(\lambda )\cdot \text{rand}(-0.5,0.5).$$ (9)

### 2.3 Preprocessing Algorithms

A literature search was done on hyperspectral imaging for medical applications, which resulted in almost 200 papers. In most of those papers, no preprocessing was mentioned. It was not always clear whether a preprocessing algorithm was used and not mentioned, or no preprocessing algorithm was applied. From the remaining papers, we identified 11 preprocessing algorithms, of which we excluded two since they are not widely applicable or require additional measurements. The algorithms that were excluded were: resonant Mie scattering extended multiplicative signal correction, due to its complexity and iterative nature being only selectively implementable;^16^^,^^51^ and retrieved background correction, due to the additional measurements that need to be made to correct for the spatial deviation of the light source.^52^

The eight remaining algorithms that we discuss in this paper are: SNV, MSC, MM, MC, AUC normalization, SW normalization, FD, and SD.

#### 2.3.1 Standard Normal Variate

SNV is commonly used in chemical analysis to remove the effects of scattering from a measured spectrum.^53^ In medical hyperspectral imaging, this method was used by Baltussen et al.,^10^ Kho et al.,^11^ Li et al.,^12^ Collins et al.,^13^ Maktabi et al.,^14^ Malegori et al.,^15^ Peñaranda et al.,^16^ and Pardo et al.^54^ In the most used version of SNV, the mean of each individual reflectance spectrum ${\overset{¯}{R}}_{\mathrm{tot}}$ is subtracted from that same individual spectrum $R_{\mathrm{tot}}(\lambda )$, and the resulting values are divided by the standard deviation of the spectrum σ of that same individual reflectance spectrum:

$$R_{\mathrm{SNV}}(\lambda )=\frac{R_{\mathrm{tot}}(\lambda )-{\overset{¯}{R}}_{\mathrm{tot}}}{\sigma }.$$ (10)

#### 2.3.2 Multiplicative Scatter Correction

MSC is often used in the chemical field and food-sciences. As the name implies, this algorithm aims to minimize the effects of scattering on the obtained spectra of samples.^55^ In medical hyperspectral imaging, MSC was used by Amigo et al.,^18^ Jian et al.,^19^ and Alrezj et al.^20^ The goal of MSC is to correct for offsets and scaling in individual spectra, so they are as similar as possible to a “reference spectrum.” In general, the “reference spectrum” is taken as the mean spectrum of the entire dataset:

$$R_{\mathrm{ref}}(\lambda )=\frac{\sum_{i=1}^{m}R_{\mathrm{tot},i}(\lambda )}{m},$$ (11)

where m is the number of spectra within the dataset. For any single spectrum within the dataset, $R_{\mathrm{tot},i}$, it is assumed that it can be modeled as

$$R_{\mathrm{tot},i}(\lambda )=c+d\cdot R_{\mathrm{ref}}(\lambda ).$$ (12)

The values of c and d are obtained for each individual reflectance spectrum by fitting Eq. (12) to each individual measured spectrum $R_{\mathrm{tot},i}(\lambda )$ using mean square error minimization.^56^ The final MSC corrected spectrum is then calculated as

$$R_{\mathrm{MSC},i}(\lambda )=\frac{R_{\mathrm{tot},i}(\lambda )-c}{d}.$$ (13)

Note that MSC and SNV both correct for scaling and an offset. However, in the implemented MSC approach, the reference spectrum $R_{\mathrm{ref}}$ will change when spectra are added or removed from the database, which will in turn change and affect the shape of all spectra $R_{\mathrm{tot},i}(\lambda )$. SNV processing is done on each spectrum separately and thus is not influenced by other spectra.

#### 2.3.3 Min–max normalization

MM, as used by Halicek et al.,^21^^,^^57^ Koprowski et al.,^22^ Wu et al.,^24^ Fabelo et al.,^25^ Martinez et al.,^26^ Leon et al.,^27^ Aboughaleb et al.,^28^ Peñaranda et al.,^16^ and Luthman et al.,^29^ is an algorithm that uses the minimum and maximum values of each individual measured reflectance spectrum $R_{\mathrm{tot}}(\lambda )$ to scale and offset that same individual spectrum:

$$R_{\mathrm{MM}}(\lambda )=\frac{R_{\mathrm{tot}}(\lambda )-\min(R_{\mathrm{tot}}(\lambda ))}{\max(R_{\mathrm{tot}}(\lambda ))-\min(R_{\mathrm{tot}}(\lambda ))}.$$ (14)

#### 2.3.4 Mean centering

MC, as used by Lasch and Noda.^30^ and Morais et al.,^31^ is aimed at reducing the effect of unwanted offsets to the signal. MC is defined as

$$R_{\mathrm{MC}}(\lambda )=R_{\mathrm{tot}}(\lambda )-{\overset{¯}{R}}_{\mathrm{tot}},$$ (15)

where the mean reflectance value of each individual spectrum ${\overset{¯}{R}}_{\mathrm{tot}}$ is subtracted from that same individual spectrum $R_{\mathrm{tot}}(\lambda )$.

#### 2.3.5 Area under the curve

AUC normalization, as used by Lu et al.,^32^^,^^33^ Kumashiro et al.,^34^ Ma et all.,^35^ Waterhouse et al.,^36^ and Leitner et al.,^37^ divides each measured spectrum $R_{\mathrm{tot}}(\lambda )$ by the AUC of that same individual spectrum:

$$R_{\mathrm{AUC}}(\lambda )=\frac{R_{\mathrm{tot}}(\lambda )}{\sum_{\lambda =\lambda_{\text{begin}}}^{\lambda_{\text{end}}}R_{\mathrm{tot}}(\lambda )}.$$ (16)

Note that since reflectance values in hyperspectral imaging are always positive, this algorithm is equal to l1-normalization as used by Wirkert et al.^58^

#### 2.3.6 Single wavelength

SW scaling, as used by Lu et al.^38^ and Baltussen et al.,^1^ is an algorithm in which each spectrum is divided by its value at a reference wavelength $\lambda_{0}$:

$$R_{\mathrm{SW}}(\lambda )=\frac{R_{\mathrm{tot}}(\lambda )}{R_{\mathrm{tot}}(\lambda_{0})}.$$ (17)

A reference wavelength is chosen in a wavelength range where absorption is minimal. This low absorption wavelength can differ between tissue types, but in tissue applications a wavelength between 700 and 800 nm is often chosen, because in that wavelength range the absorption by blood, fat, and water is low. In our analysis, we use 730 nm as $\lambda_{0}$, which was found to produce the best results.

#### 2.3.7 First derivative

In FD processing, as used by Amigo et al.,^18^ Hu et al.,^40^ Liu et al.,^39^ Peñaranda et al.,^16^ and Mellors et al.,^41^ the FD of the spectrum, instead of the spectrum itself, is used for tissue classification:

$$R_{\mathrm{FD}}(\lambda )=\frac{dR_{\mathrm{tot}}(\lambda )}{d\lambda }.$$ (18)

FD processing results in a characterization of the localized slope of the spectrum. FD processing is very sensitive to noise. Therefore, in general, filtering or smoothing of the spectrum is performed before FD processing, such as Savitzky–Golay filtering.^16^^,^^18^^,^^39^^–^^41^ In our analysis, we used Savitzky–Golay filtering and optimized the window size so it would result in the lowest overlap coefficient, which was a window size of 199 nm.

#### 2.3.8 Second derivative

SD processing, as used by Wang et al.^42^ and Zheng et al.,^43^ uses the SD of the spectrum for tissue classification:

$$R_{\mathrm{SD}}(\lambda )=\frac{d^{2}R_{\mathrm{tot}}(\lambda )}{d\lambda^{2}}.$$ (19)

SD processing results in a characterization of the rate with which the slope of the spectrum changes. Similar to FD processing, filtering or smoothing of a spectrum is performed before SD processing. In our analysis, we used again the Savitzky–Golay filtering and optimized the window size so it would result in the lowest overlap coefficient, which was a window size of 199 nm.

### 2.4 Data Analysis

#### 2.4.1 Overlap coefficient

To quantify the effectiveness of each preprocessing algorithm, we use the Szymkiewicz–Simpson coefficient or overlap coefficient, O, which is defined as the number of reflectance values in the intersection of two sets of spectra, divided by the number of reflectance values in the smallest set:^59^

$$O(\lambda )=\frac{\left|R_{1}(\lambda )\cap R_{2}(\lambda )\right|}{\min(|R_{1}(\lambda )|,|R_{2}(\lambda )|)}.$$ (20)

The overlap coefficient is a similarity measure for two sets of spectra, giving the overlap between the sets as a value between 0 and 1 for each wavelength.

**Fig. 3:**
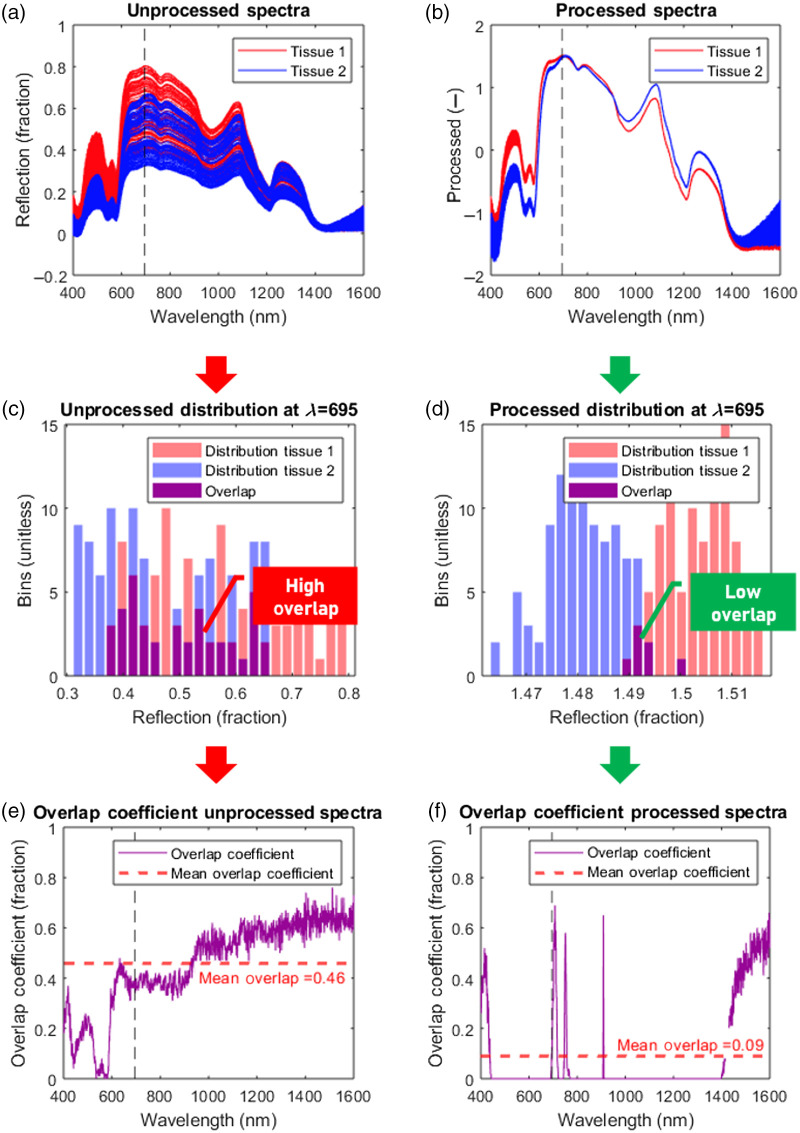
Calculation of the overlap coefficient for (a) unprocessed synthetic spectra and (b) preprocessed synthetic spectra. In the unprocessed spectra, large nontissue-specific variations in the spectra are visible, which make it difficult to distinguish tissue 1 (red lines) from tissue 2 (blue lines). In the processed spectra, the nontissue-specific variations are reduced significantly. (c), (d) To calculate the overlap coefficient per wavelength histograms of the reflectance values at a single wavelength are created. The overlap coefficient is equal to the number of reflectance values in the intersection of the two sets (purple bars), divided by the number of reflectance values in the smallest set [Eq. (20)]. (e), (f) The mean overlap coefficient over all wavelengths is calculated (dashed red line). This shows that the unprocessed spectra have a high overlap, whereas the processed spectra have a low overlap.

Figure 3 shows how the overlap coefficient is calculated. Two types of tissues are simulated [Fig. 3(a)], where one type has a BVF of 0.5% (red lines) and the other has a BVF of 2% (blue lines). For each tissue type, 100 spectra were created with variable amounts of glare and height differences. In Fig. 3(b), the effect of preprocessing is shown on the spectra. In the unprocessed spectra [Fig. 3(a)], large nontissue-specific variations can be seen. In the processed spectra [Fig. 1(b)], these nontissue-specific variations are reduced and the spectra can be better distinguished from each other. To quantify how well the spectra can be distinguished from each other, the overlap coefficient is used. First, the reflectance values for a single wavelength are taken from two sets of spectra and a histogram is created with a total of 25 bins for both spectra combined [Figs. 3(c) and 3(d)]. The number of spectra in the intersection (purple bars) are then divided by the number of spectra in the smallest set (which is equal to 100 for the synthetic spectra). The overlap coefficient is calculated for each wavelength of the spectra for both the unprocessed [Fig. 3(e)] and the processed spectra [Fig. 1(f)]. The mean is then taken over all wavelengths, resulting in the mean overlap coefficient (dashed red line). As shown in Fig. 3, the mean overlap coefficient is a similarity measure between the types of tissue, where less similarity means that the two types of tissue are more distinguishable from each other.

To assess general trends, the improvement in the overlap coefficient for each preprocessing algorithm $O_{\text{improvement}}$, is calculated as

$$O_{\text{improvement}}=\frac{O_{\text{unprocessed}}-O_{\text{processed}}}{O_{\text{unprocessed}}}\cdot 100\%,$$ (21)

where $O_{\text{unprocessed}}$ is the average overlap coefficient over all wavelengths for the two sets before any preprocessing algorithm is applied and $O_{\text{processed}}$ is the average overlap coefficient over all wavelengths between the two sets after a specific preprocessing algorithm is applied.

### 2.5 Clinical Data

To test whether our results on synthetic data can be translated to clinical applications, we also investigated the overlap coefficient for different preprocessing algorithms on two clinical datasets from colorectal cancer patients^10^ and from breast cancer patients.^11^ Both datasets were collected previously using a bench-top system and contain influence of glare and height differences. A detailed description of the materials and methods can be found in these papers, but a short description will be provided here. Both studies were performed on *ex-vivo* tissue samples with bench-top hyperspectral systems. Each pixel within a hyperspectral image was correlated to histopathology to determine the tissue type within the pixel. Data were obtained from Baltussen et al.,^10^ who imaged tissue slices from resected colorectal tissue from 32 patients with two hyperspectral line scanning camera (Spectral Imaging Ltd., Finland) in the visual (PFD-CL-65-V10E) and the near-infrared (VLNIR CL-350-N17E) range (400 to 1600 nm with an average resolution of 4 nm). 2170 spectra were present in the combined dataset, of which 857 from fat, 563 from muscle, and 750 from tumor tissue. Using the same setup, Kho et al.^11^ imaged lumpectomy specimens and slices from resected breast tissue. Only the lumpectomy data were considered for this paper due to the reduced effect of height differences in the slices dataset. From the lumpectomy dataset, the spectra of eight patients were included, which gave a total of 1072 spectra containing 453 spectra of healthy breast tissue and 619 tissue of tumorous breast tissue, which included both ductal carcinoma *in situ* and invasive carcinoma. Please note that due to the use of two hyperspectral cameras in both setups, the preprocessing was done for each camera separately, meaning that the data from the visual spectra were processed separately from the infrared spectra.

A difference between the clinical and synthetic datasets lies in the variations that are included in them. First of all, in our simulations, we compared a dataset where all the spectra had a BVF of 2% to a dataset where all the spectra had a BVF of 5%. However, in reality, the difference in healthy and tumor tissues will not be such a clear dichotomy—both tissue types will have distributions of BVFs that most likely overlap. Second, while the synthetic dataset considers only one changing optical property, a clinical dataset will include variation of multiple optical properties at the same time. To illustrate both effects, we performed simulations with optical properties from a study with diffuse reflectance spectroscopy in the breast, which has shown that there are indeed large variations in optical properties between tissue types^60^^,^^61^ (note that DRS is a contact measurement and does not have any effect from glare or distance). Based on the optical properties they measured, we performed simulations to illustrate how these variations influence the effect of preprocessing algorithms.

The main contrast for healthy and tumor tissue in breast comes from the fat-water ratio, but as can be seen in Table 2, many parameters have a large variation within each tissue type. We made spectra by combining random values for each optical property that lie within the ranges given in Table 2. In total 2000 spectra were simulated, of which 1000 for healthy and 1000 for tumor. The spectra are created by choosing optical properties within the range specified in the table for each spectra and tissue type. The simulated spectra using the optical properties as stated above gives us two distributions of spectra belonging to simulated healthy tissue and simulated breast tumors. For the overlap coefficient a bin size of 50 was chosen.

**Table 2:** Tissue parameters used to create the synthetic data, based on the DRS data from de Boer et al.^60^^,^^61^ The numbers in the table indicate the ranges are given that were used in our simulated spectra. In total, 2000 spectra were simulated, of which 1000 for healthy and 1000 for tumor. The spectra were made by choosing optical properties within the range specified in the table for each spectra and tissue type.

	BVF (%)	Saturation (%)	WVF (%)	FVF (%)	Scatter amplitude	Scatter slope
Simulated healthy	0 to 6	30 to 80	2 to 4	30 to 68	10 to 15	0.8 to 1
Simulated tumor	2 to 10	5 to 60	35 to 37	33 to 35	12 to 35	0.9 to 1.3

## 3 Results

### 3.1 Synthetic Spectra

**Fig. 4:**
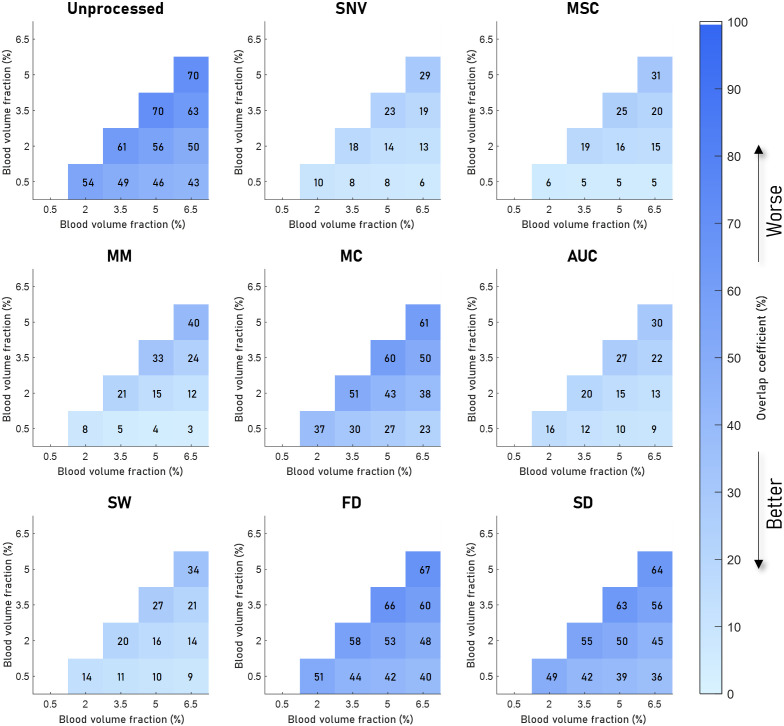
Overlap between sets of spectra with different BVFs for eight preprocessing algorithms. The color and the number indicate the overlap coefficient, where a higher overlap coefficient indicates that more of the spectra overlap and would be worse for classifying. The mean overlap coefficient is shown for five different BVFs, where the unprocessed result is shown in the top left image, and the various preprocessing techniques in the rest of the image. Here, it can be seen that SNV and MSC result in a low overlap. AUC, SW, and MM have similar overlap but higher than SNV and MSC. MC, FD, and SD have significantly higher overlap after processing, with FD and SD having the highest.

**Fig. 5:**
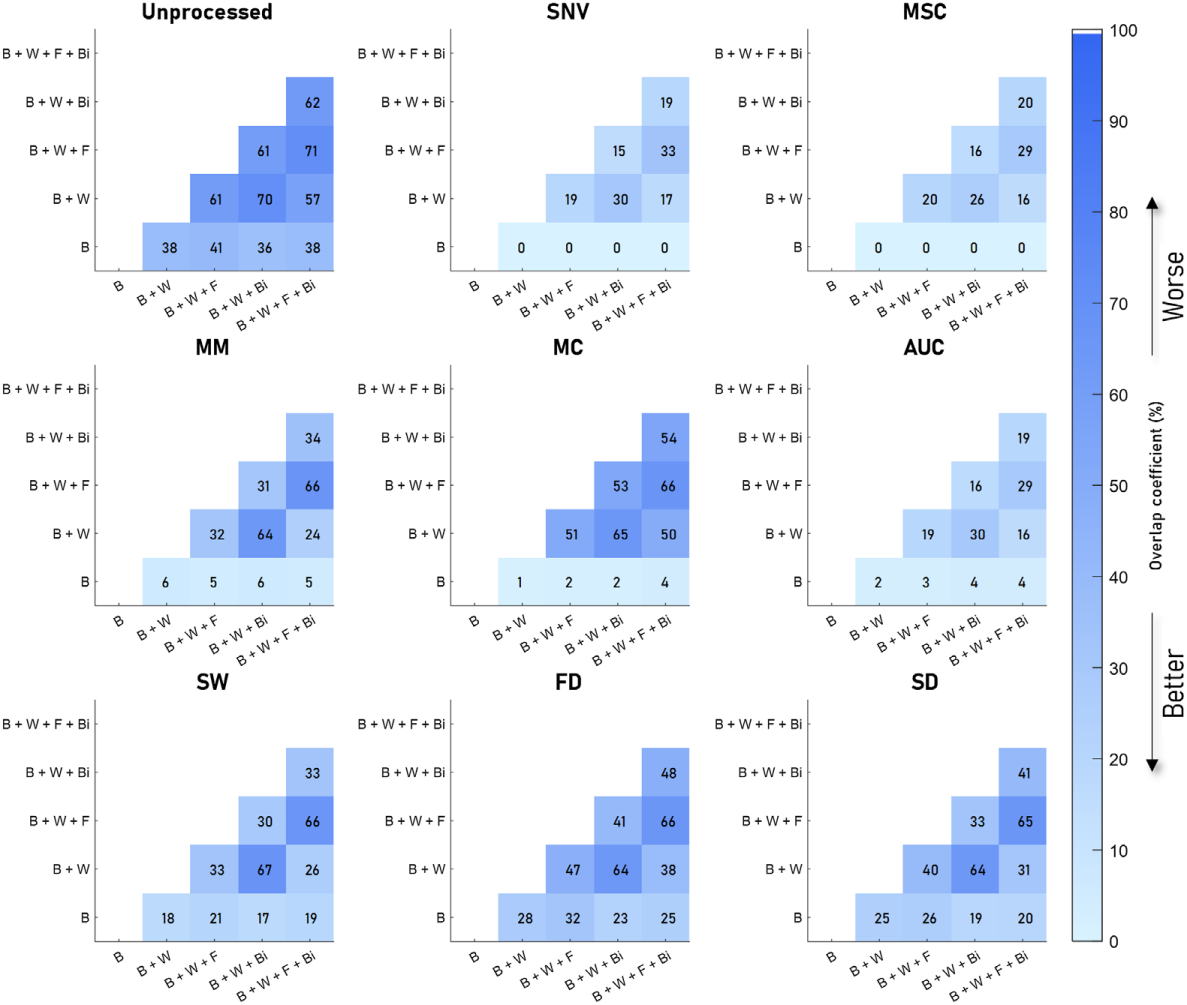
Overlap between sets of spectra with different absorbers for eight preprocessing algorithms. The axes are labeled with the type of absorbers included, where B, blood; W, water; F, fat; and Bi, bilirubin. SNV, MSC, and AUC reduce the overlap the most, followed by MM, which reduces the overlap less than SNV, MSC, or AUC but still reduces the overlap significantly. MC reduces the overlap less. Finally, FD, SD, and SW reduce the overlap the least with some high values for a few combinations remaining.

**Fig. 6:**
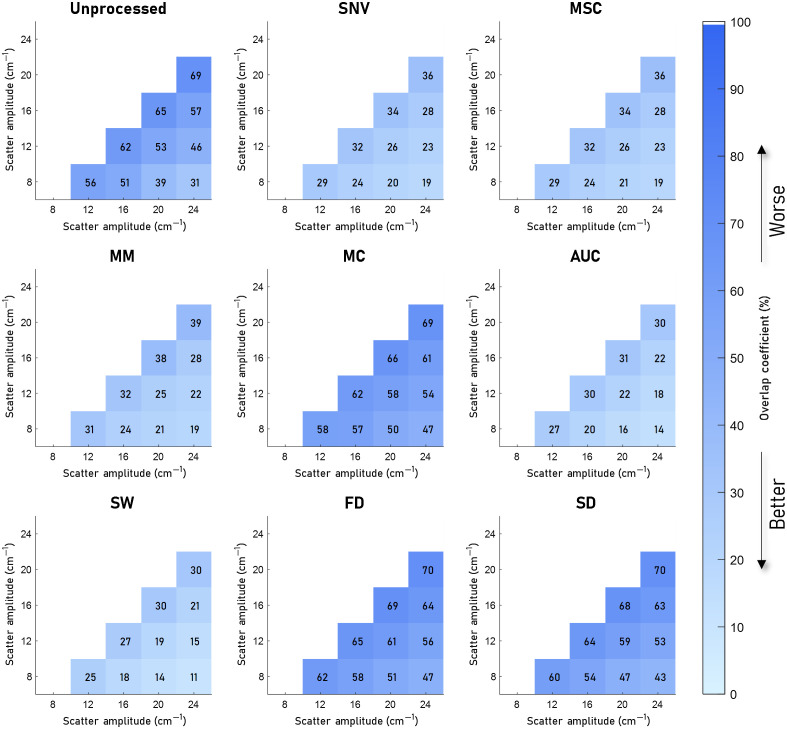
Overlap between sets of spectra with different scatter amplitudes (a) for eight preprocessing algorithms. The mean overlap coefficient is shown for five different values of a. The unprocessed result is shown in the top left image, here a high overlap due to the nontissue-specific variations can be seen between the different values of a. AUC and SW reduce the overlap significantly. MM, SNV, and MSC remove the overlap less than AUC and SW. MC, FD, and SD reduce the overlap the least.

**Fig. 7:**
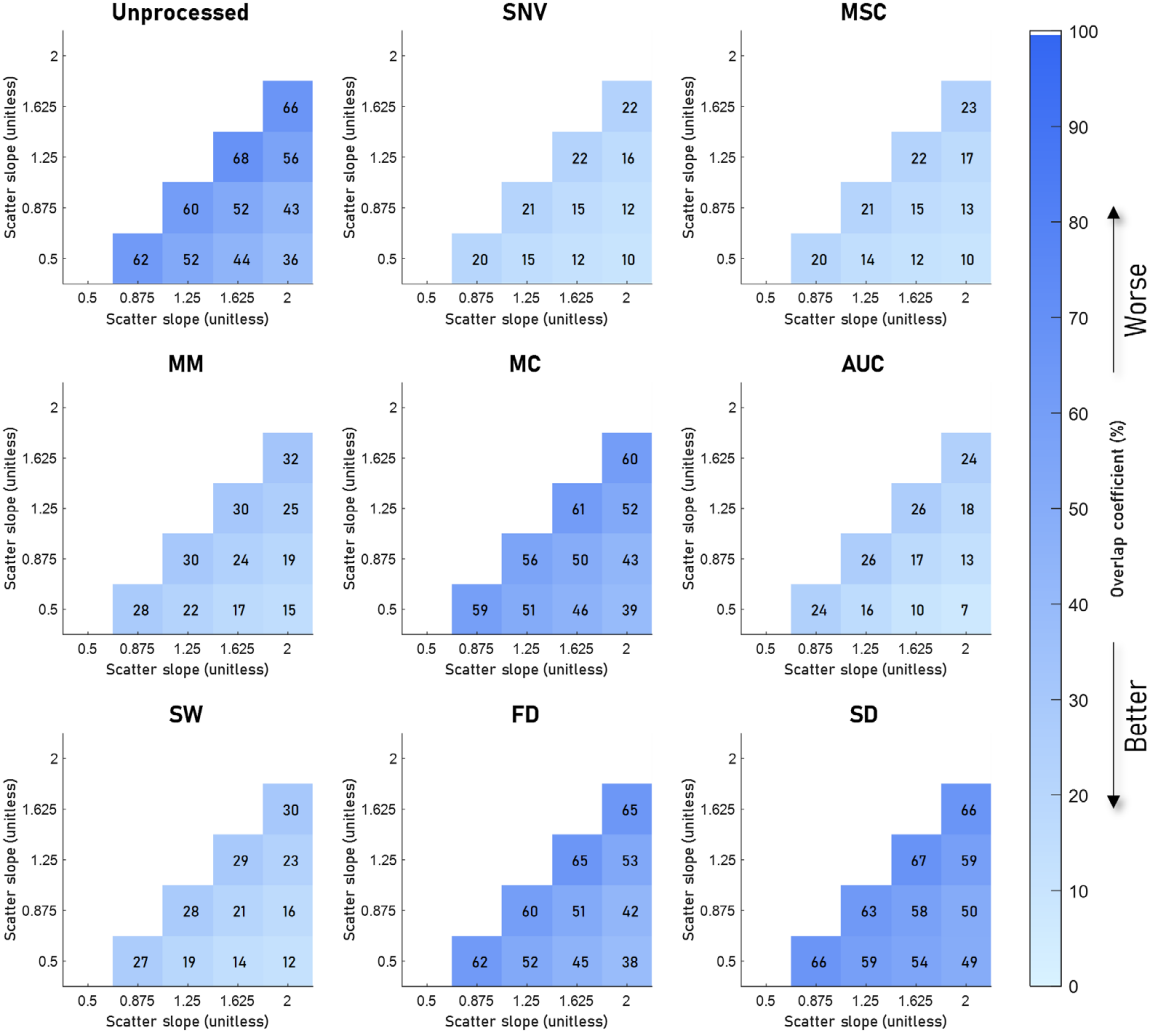
Overlap between sets of spectra with different scatter slopes (b) for eight preprocessing algorithms. The mean overlap coefficient is shown for five different values of b. The unprocessed result is shown in the top left image, here a high overlap due to the nontissue-specific variations can be seen between the different values of b. Here, SNV, MSC, and AUC reduce the overlap the most. SW and MM perform comparably with SW reducing the overlap only slightly more. MC, FD, and SD reduce the overlap the least, with SD increasing the overlap.

Figures 4–7 visualize the results of our analysis for the synthetic datasets, where we varied the BVF (Fig. 4), the presence of different absorbers (Fig. 5), the scatter amplitude (Fig. 6), and the scatter slope (Fig. 7). Each block within a figure represents the results for the unprocessed spectra or spectra processed with the algorithm. Each square represents the mean overlap coefficient between two sets of spectra with different tissue properties, where the properties of the sets are specified on the horizontal and vertical axes. A lighter square indicates a lower overlap coefficient and thus a better performance of the preprocessing algorithm. Overall, as expected, in the unprocessed spectra (upper left corner) the overlap between sets of spectra decreases when the differences in optical properties increase.

The results of the analysis on the synthetic data with different BVFs are visualized in Fig. 4. As shown in the top left panel, the mean overlap coefficients between the different BVF spectra of the unprocessed spectra are high (on average). Clearly, SNV and MSC preprocessing result in the lowest overlap coefficients (on average 15%), whereas FD and SD show the highest overlap coefficients (average 53% and 49%).

The results of the analysis on the synthetic data with adding absorbers water (W), fat (F), and bilirubin (Bi) to blood (B) are visualized in Fig. 5. Here, the type of absorbers is shown on the horizontal and vertical axes. The least overlap is seen after AUC, SNV, and MSC, which reduce the overlap between the spectra with different absorbers, resulting in average overlap coefficients of 13%, 13%, and 14%, respectively.

The results of the analysis on the synthetic data with different scatter amplitudes are visualized in Fig. 6. For the scatter amplitude, AUC and SW reduce the overlap the most, resulting in an average overlap coefficient of 23% and 21%, respectively; MC, FD, and SD again reduce the overlap the least, with an overlap coefficient of 58%, 60%, and 58%, respectively, which is worse than no preprocessing

The results of the analysis on the synthetic data with different scatter slopes are visualized in Fig. 7. Here, AUC, SNV, and MSC reduce the overlap the most, resulting in an average overlap coefficient of 17%, 17%, and 18%, respectively. The algorithms that reduced the overlap the least were MC, FD, and SD, with average overlap coefficients of 52%, 53%, and 60%, respectively.

### 3.2 Clinical Data

The results are presented in the same manner as the synthetic data, but the horizontal and vertical axes indicate the tissue type to which the spectra belong instead of the tissue parameters shown in the synthetic data.

#### 3.2.1 Colon

**Fig. 8:**
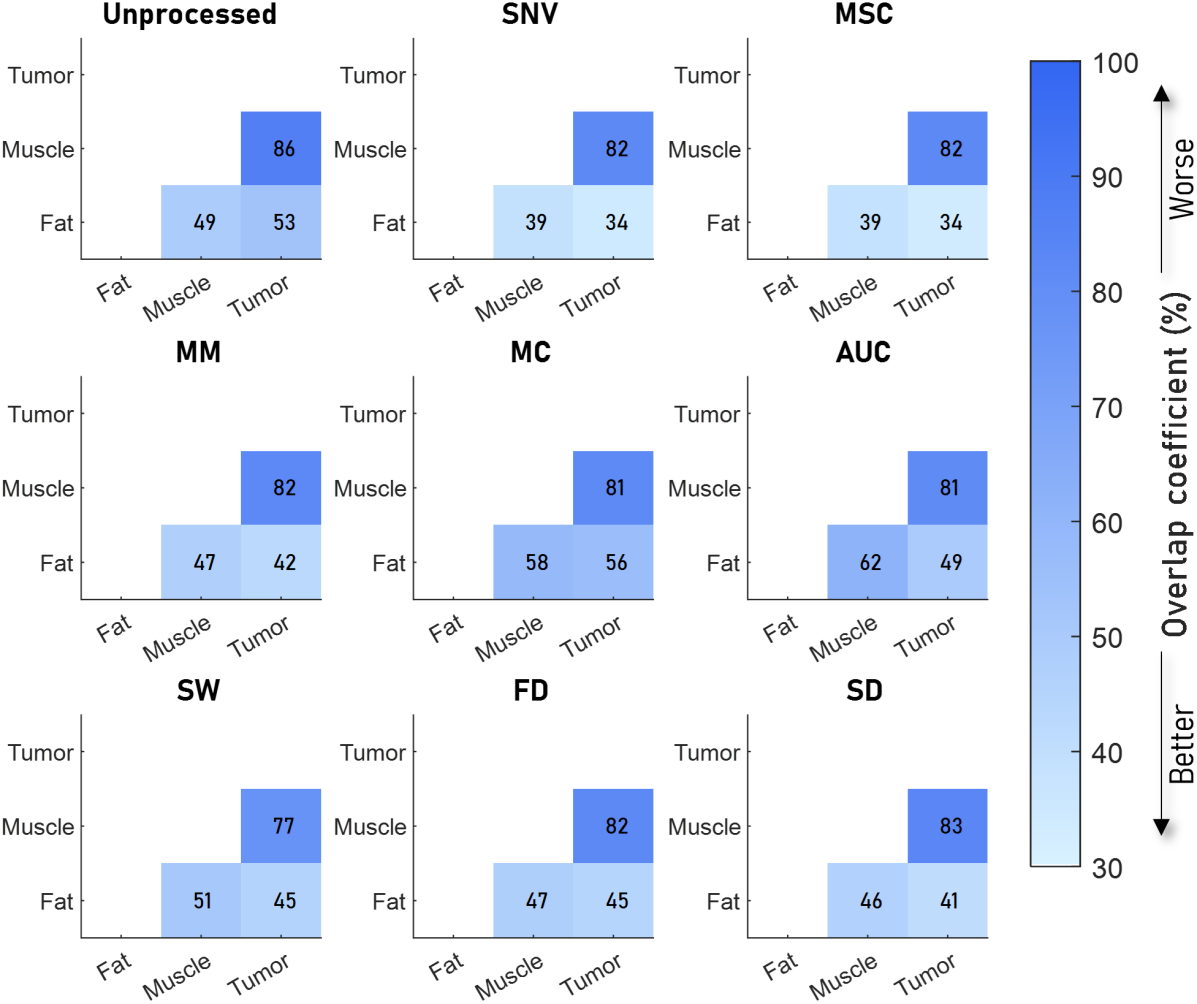
Overlap between sets of spectra from different tissue types in colon samples measured by Baltussen et al.^10^ for eight preprocessing algorithms. The unprocessed result is shown in the top left image. SNV and MSC processing resulted in the lowest sum of overlap coefficients, whereas MC and AUC resulted in the highest overall overlap coefficients. SW produces the lowest overlap between tumor and muscle.

The colon data were divided into three categories, muscle, fat, and tumor. In all cases, the overlap between spectra from tumor and muscle tissue was much higher than the overlap between tumor and muscle tissue compared with fat (Fig. 8). Most preprocessing algorithms reduce the overlap coefficient compared with the unprocessed spectra. However, AUC and MC increase the overlap coefficient for fat versus muscle. Overall, SNV and MSC resulted in the lowest overlap coefficients, whereas MC and AUC resulted in the highest overlap coefficients. SW produces the lowest overlap between tumor and muscle.

#### 3.2.2 Breast

**Fig. 9:**
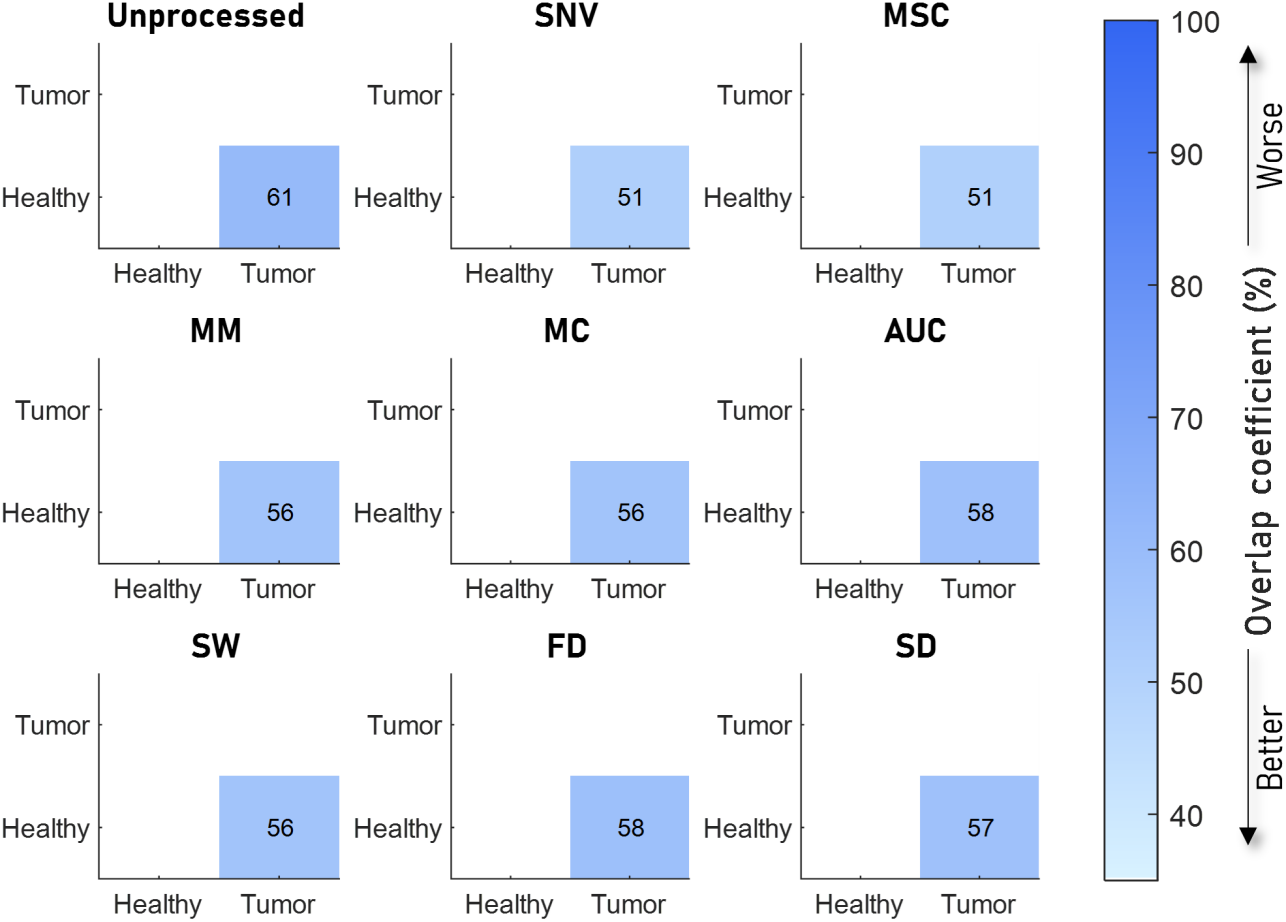
Overlap between sets of spectra from different tissue types in breast samples measured by Kho et al.^11^ for eight preprocessing algorithms. The unprocessed result is shown in the top left image. SNV and MSC processing resulted in the lowest overlap coefficients, whereas AUC and FD resulted in the highest overall overlap coefficients.

For the breast data, the tissue was divided into two categories: healthy and tumor tissues. Therefore, we only obtained a single value for the overlap for each algorithm (Fig. 9). All algorithms reduced the overlap coefficient compared with unprocessed data. SNV and MSC resulted in the lowest overlap coefficients.

#### 3.2.3 Simulated mixed tissue

**Fig. 10:**
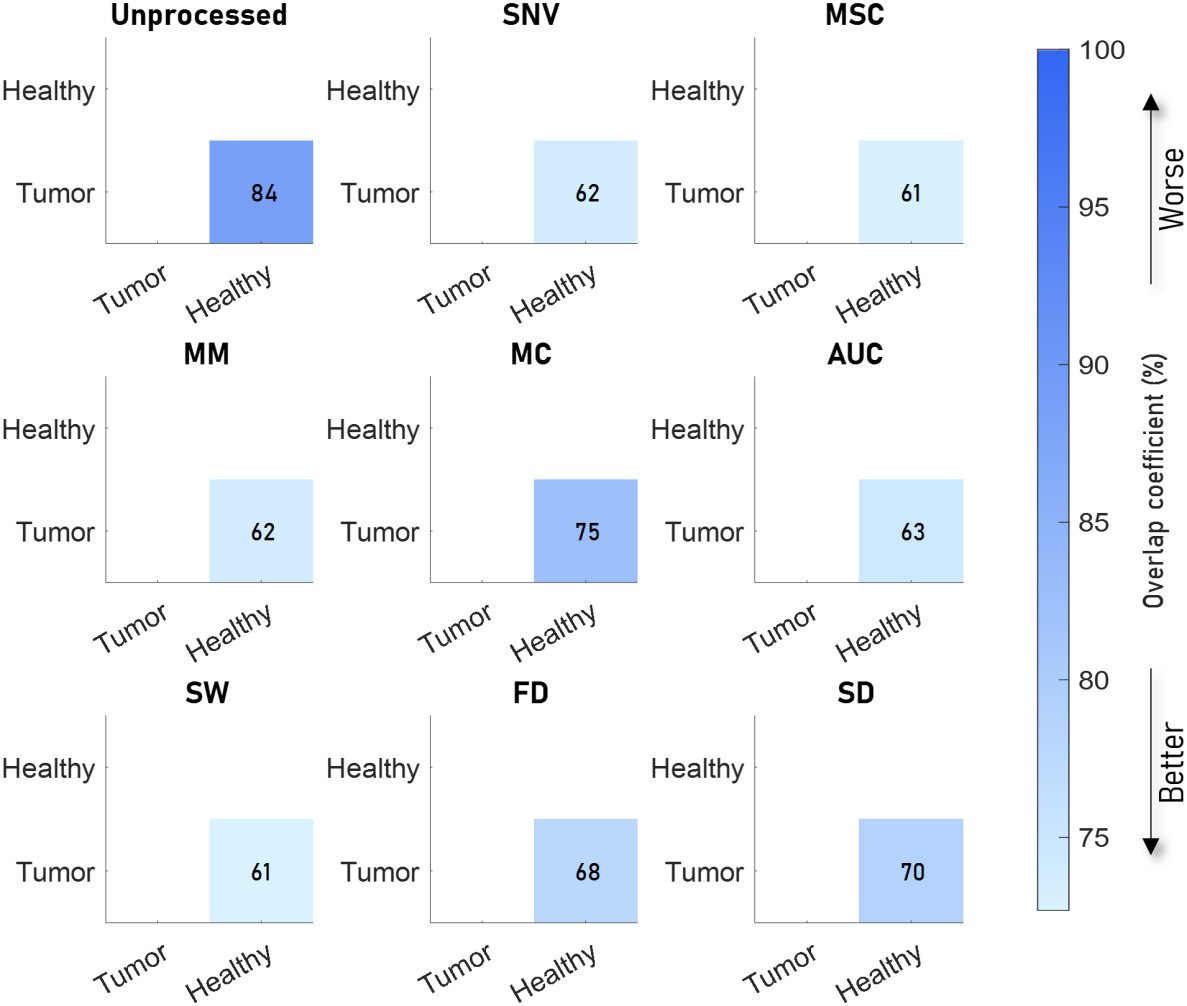
Overlap between sets of spectra from mixed tissue types, which includes the variation between and within patients. The overlap is lowest for SNV, MSC and SW and highest for MC, FD and SD.

The results of the mixed tissue type that includes the variations discussed are shown in Fig. 10. In the simulated data, SNV, MSC, and SW reduce the overlap the most, whereas MC, FD, and SD perform the worst.

**Fig. 11:**
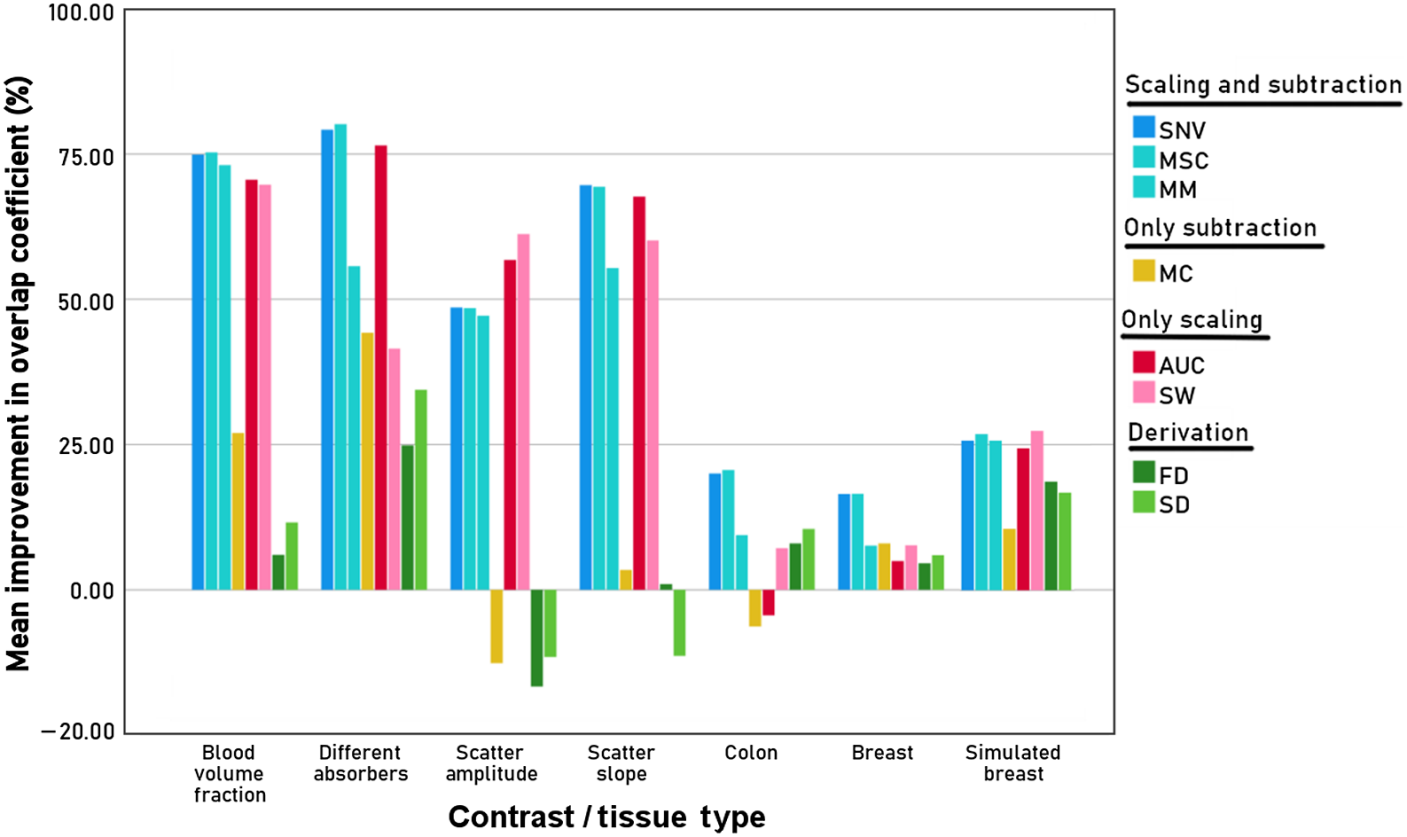
Summary of the data presented in Figs. 4–10. For the four types of synthetic data with different tissue contrast (different absorbers, BVF, scatter amplitude, and scatter slope) as well as the colon and breast data; and the simulated breast data, the mean improvement in overlap coefficient relative to the unprocessed data is depicted per preprocessing algorithm. Preprocessing algorithms from the same category have a similar color. For the colon, the improvement in the overlap coefficient shown is the average of the improvement for each of the combinations of tissue classes.

To facilitate easier comparison of the different algorithms for the tissue types and the clinical data, the mean improvement in overlap coefficient relative to the unprocessed data is shown in Fig. 11. The first four columns show the improvement for the synthetic spectra and the last two columns show the results for the clinical data. For the colon, the improvement in the overlap coefficient shown is the average improvement for all three classes of tissue (fat, muscle, and tumor). For the synthetic data, SNV and AUC have the highest overall improvement in the overlap coefficient. Algorithms that correct for scaling and an offset (blue bars; SNV, MSC, and MM) have the highest improvement in the overlap coefficient for the BVF, different absorbers, and scatter slope. MM generally performs less well compared to MSC and SNV. Algorithms that correct for scaling alone have the highest improvement for the scatter amplitude (red bars; AUC and SW). The two algorithms that use the derivate of the spectrum (green bars; FD and SD) and the algorithm that only subtracts an offset (MC) have a much smaller decrease in the overlap coefficient compared with all the other algorithms.

For the clinical data, the improvement in the overlap coefficient is less pronounced. For both the breast and colon tissues, similar trends can be observed as in the synthetic data, where the algorithms that correct for scaling and an offset (blue bars) and the algorithms that correct for scaling alone (red bars) perform better than the algorithms that only correct for an offset (yellow bar) and use differentiation (green bars). For the colon and the breast, MSC and SNV perform the best. For the colon, MC and AUC increase the overlap between the spectra from different tissue types. For the simulated mixed breast data, the improvement in overlap is lower than for the synthetic data and closer to the clinical data. Again, similar trends hold for the simulated breast data, where both the scaling and subtraction techniques (blue bars), and the only scaling techniques (red bars) perform well in reducing the overlap.

## 4 Discussion

In this paper, we compared the suitability of preprocessing algorithms to reduce the nontissue-specific spectral variations caused by glare and sample thickness variations in a simulated dataset resembling diffuse reflection spectra from human tissue, and in a clinical dataset containing hyperspectral images of breast and colon tissues. Overall, our results indicate that preprocessing algorithms can reduce the unwanted spectral variations caused by variations in glare and sample thickness while retaining contrast due to differences in tissue optical properties.

Glare and sample thickness variations introduce both an offset and a multiplication. Therefore, it was expected that algorithms that subtract an offset and divide by a scaling factor would perform the best. SNV, MSC, and MM determine an offset and scaling factor for each individual spectrum, thereby reducing the influences of glare and sample thickness variations. In general, the algorithms that subtract an offset and divide by a scaling factor (SNV, MSC, and MM) perform the best, except for tissue contrast related to the scatter amplitude. Since glare and sample thickness variations introduce both an offset and a multiplication, it was expected that SNV, MSC, and MM reduce these variations the best. Since SNV and MSC were originally developed to reduce the effects of scattering, it was interesting that SNV and MSC do not only perform well for tissue contrast related to absorption but also related to the scatter slope. SNV and MSC produce very similar results, and MM generally performs slightly worse. Algorithms that only divide by a scaling factor but do not correct for an offset (AUC and SW) also perform well but slightly less than the above-discussed algorithms that also subtract an offset before dividing by a scaling factor. The algorithm that only removes the offset (MC) performs poorly on reducing the overlap coefficient, which can be explained by the fact that it does not reduce variations as a result of variations in the sample thickness, leaving overlap in the spectra as can be seen in the Supplementary Material. The two algorithms that use the derivative of the spectrum (FD and SD) perform significantly worse for all types of tissue contrast compared with the other algorithms. We had expected that FD and SD would also perform well since the derivatives of the spectra are less influenced by offsets and scaling factors. In the Supplementary Material, it can be seen that FD and SD are less able to remove the influence of glare and sample thickness variations compared with other preprocessing algorithms and also reduce the contrast between the spectra.

The general trends observed in the synthetic datasets translated well to the clinical datasets. The improvement in overlap coefficient is lower for the clinical dataset compared with the synthetic dataset, but this is to be expected, because in the clinical spectra the natural variation in tissue structure and composition in samples from the same tissue type is incorporated—not every tumor is the same. For example, in our synthetic dataset, we compare two “tissue types” where one has a BVF of 2% and the other of 5%. Even if on average, the BVF of healthy tissue is 2% and of tumor tissue 5%, most likely in reality the ranges of BVFs for both tissue types overlap. To verify that the heterogeneity between and within patients can explain the lower overlap coefficients in clinical datasets, we have simulated spectra with additional variations in optical properties within each tissue type. The results for the simulated breast spectra are comparable with the clinical breast data, most importantly, as was the goal for the simulated breast tissue data, it shows a clear reduction in the effectiveness of all preprocessing techniques due to the variations mentioned above. The general trends observed in the synthetic datasets translated well to the clinical dataset, except the performance of AUC in the colon dataset, where it resulted in worse overlap coefficients compared with the original spectra without preprocessing.

Our results can be useful for the development of classification algorithms in a dataset with variations in glare and sample thickness. For example, De Boer et al.^60^^–^^62^ found for breast cancer that the fat/water ratio could discriminate between healthy and tumor tissues with an accuracy of 100% and that the fat/water ratio in breast tumor tissue was close to zero due to the absence of fat in tumor tissue, indicating that the contrast is based on the presence of additional absorbers. For this type of contrast, MSC, SNV, and AUC perform the best (Fig. 11). For the clinical breast dataset, MSC and SNV indeed perform the best, whereas AUC performs worse.

For the colon, three tissue types are classified, which makes it more complex to analyze. When analyzing multiple classes of spectra, two approaches can be taken when encountering such a problem. First, one can identify which two classes are most important to distinguish (e.g., muscle versus tumor) and choose the algorithm based on the contrast between those two classes. A second approach would be to first calculate which tissue types have the highest overlap coefficient. The type of tissue contrast that gives the highest overlap, and thus the lowest distinguishability, should then be the leading choice for a preprocessing algorithm. For the colon data, we are most interested in muscle versus tumor, where we expect differences in BVF and scattering.^63^^–^^66^ Based on our synthetic data, SNV, MSC, and SW would be good candidates, which corresponds to our findings for the clinical dataset.

We did not directly test how the different preprocessing algorithms influence tissue classification algorithms since many different types of classification algorithms can be developed. Nevertheless, the quality of the data used to develop an algorithm is known to influence the quality of the developed algorithm. Therefore, preprocessing is often used for classification algorithms. For the development of a tissue classification algorithm, removing variations in the data not directly related to differences between tissue types is expected to improve the accuracy of the developed algorithm. Since glare and sample height differences are not discriminating features between tissue types, reducing unwanted spectral variations due to distance and glare while maintaining contrast between tissues with different optical properties should improve tissue classification algorithms.

Based on our results, for the development of tissue classification algorithms in a dataset with variations in glare and sample thickness, we do not recommend the use of MC, FD, and SD as preprocessing algorithms since they performed much worse compared to the other algorithms. Because SNV and MSC normalization perform equally well, it might not be useful to test both algorithms on the same dataset. Furthermore, since the MSC normalization of an individual spectrum depends on the other spectra within the dataset, individually processed spectra will change when new spectra are added to the dataset. Thus, for large datasets, the performance of four preprocessing algorithms could be investigated: SNV, MM, AUC, and SW. A power analysis for four algorithms could result in a larger dataset than practical for a study. If the contrast between tissue types is unknown, the best choice for a preprocessing algorithm would then be AUC or SNV since on average these outperform the other algorithms for the four types of contrast investigated in this paper. If the contrast between tissue types is known, we recommend the following preprocessing algorithms: SNV for contrast due to changes in BVF; AUC or SNV for contrast due to changes in the type of absorbers in the tissue; AUC or SW for contrast due to changes in the scatter amplitude; and AUC or SNV for contrast due to changes in the scatter slope.

In *ex-vivo* settings with benchtop systems, the influences of glare and distance differences can be reduced by polarization filters and surface profilometry. For *in vivo* applications, this would hamper real-time feedback. Since classification algorithms have to be developed on similar data as the data that will be acquired during its clinical application, it is essential that *ex-vivo* studies that are performed to develop classification algorithms for *in vivo* applications use the same preprocessing algorithms (and thus not polarization filters and profilometry).

## 5 Conclusion

This paper provides researchers with a solid basis to identify the most suitable preprocessing algorithm that decreases variation due to glare and sample thickness in spectra while maintaining as much contrast between tissue types as possible. We compared eight commonly used preprocessing algorithms and identified four algorithms that we found suitable to use before developing an algorithm for tissue classification: SNV, MM, AUC, and SW. For very large datasets, all four algorithms can be tested, but for smaller datasets, we recommend to choose one or two algorithms *a priori*, based on the expected contrast between tissue types.
