# Supplementary material for: Comparison of preprocessing techniques to reduce nontissue-related variations in hyperspectral reflectance imaging
Source: J Biomed Opt. 2022 Oct 7;27(10):106003. doi: 10.1117/1.JBO.27.10.106003 (PMC9541333; doi:10.1117/1.JBO.27.10.106003)
Supplement: Supplementary file 1 [file JBO_027_106003_SD001.pdf]

Supplementary Material

In this supplementary material, the result of applying the algorithms on the synthetic data with different tissue parameters is shown. The resulting spectra are shown, where the overlap of these spectra represent the results seen in Figures 4 to 7 in the paper.

Standard Normal Variate

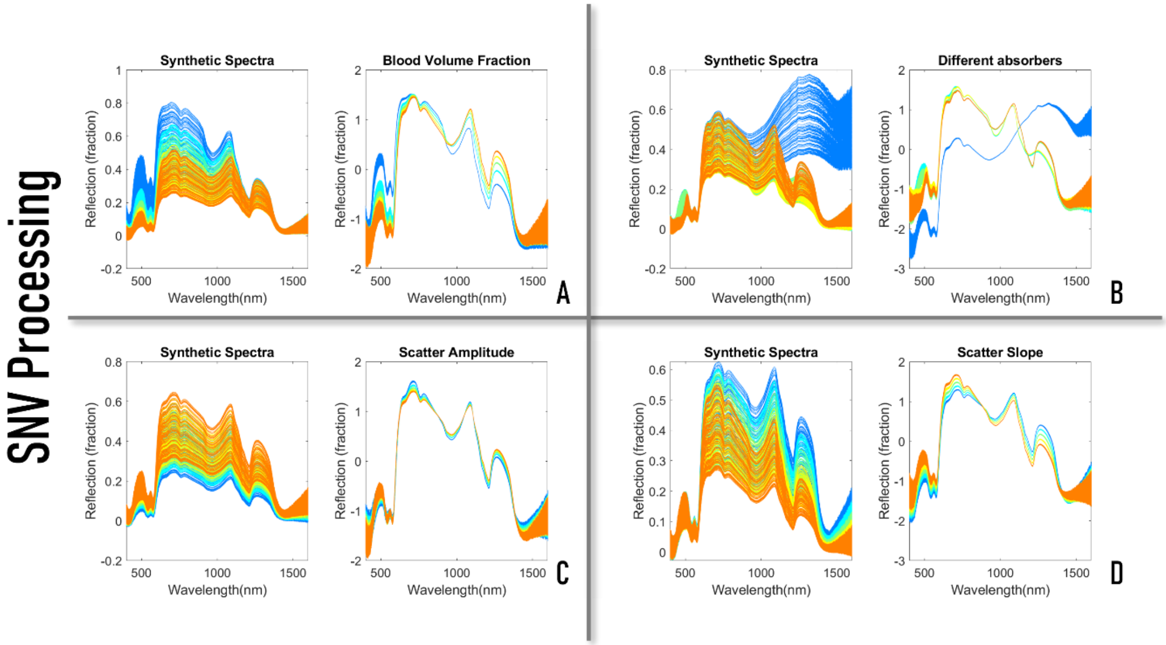

Figure S.1, Four different situations of changing parameters and the effect of SNV on the spectra. The four spectra compared are; blood volume fraction(*A*), here it can be seen that almost all the effects of glare and height differences are removed from the spectra and that significant contrast remains; different absorbers (*B*), where it can be seen that the algorithm greatly reduces the overlap between the spectra after processing; scatter amplitude (*C*), here very little contrast can be seen and the overlap is reduced less than the spectra with absorption contrast; scatter slope (*D*), again there is more overlap compared to BVF but some contrast can be seen in at the start and end of the spectra.

<u>MSC</u>

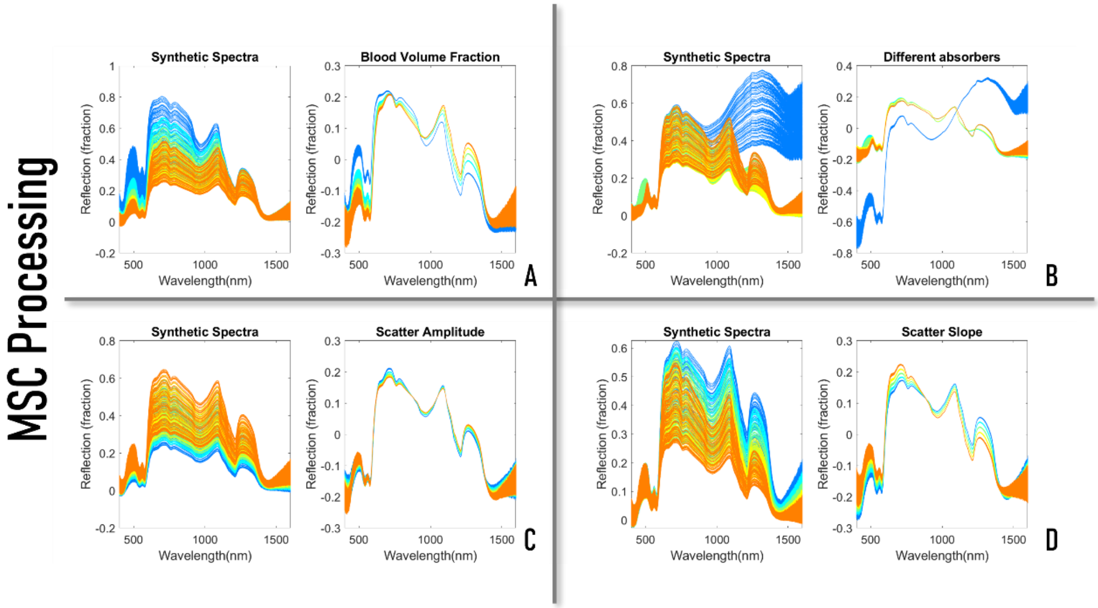

Figure S.2, Four different situations of changing parameters and the effect of MSC on the spectra. The four spectra compared are; blood volume fraction(*A*), here it can be seen that almost all the effects of glare and height differences are removed from the spectra and that significant contrast remains different absorbers (*B*), where it can be seen that the algorithm greatly reduces the overlap between the spectra after processing but flattens the spectra compared to SNV;; scatter amplitude (*C*), here almost no contrast can be seen but the overlap is reduced less than the spectra with absorption contrast; scatter slope (*D*), again there is more overlap compared to BVF but contrast can be seen in at the in the middle of the spectra similar to SNV.

Min-max

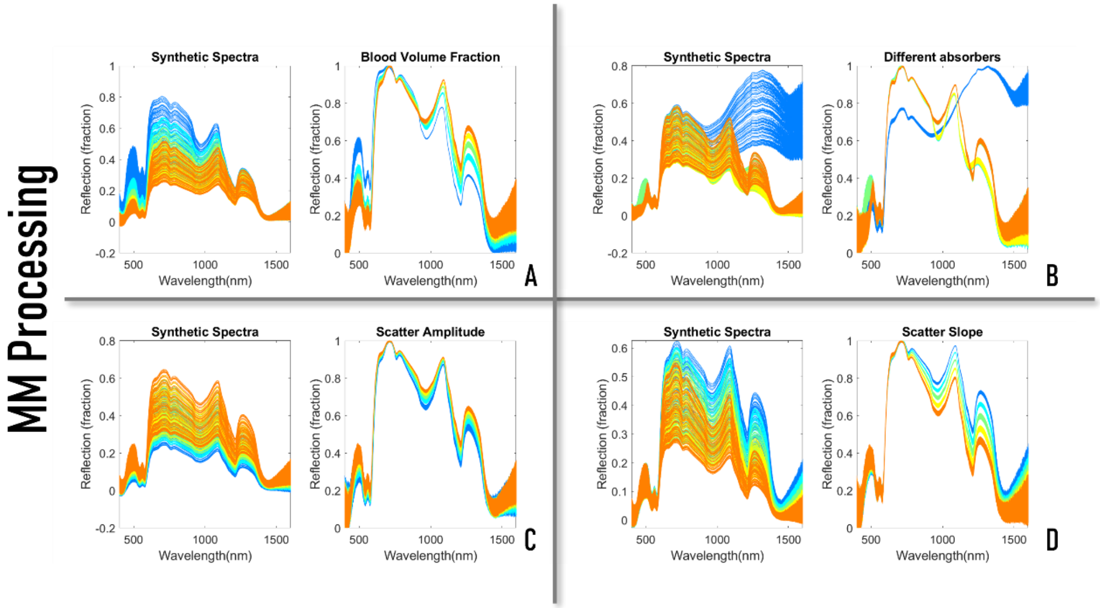

Figure S.3, Four different situations of changing parameters and the effect of MM on the spectra. The four spectra compared are; blood volume fraction(*A*), here it can be seen that almost all the effects of glare and height differences are removed, with good contrast after the maximum point in the spectra at around 800 nm; different absorbers (*B*), where it can be seen that the algorithm greatly reduces the overlap between the spectra after processing; scatter amplitude (*C*), here little contrast can be seen and the overlap is comparable to SNV and MSC; scatter slope (*D*), again there is more overlap compared to the scatter amplitude but contrast can be seen at the end of the spectra, where variation due to distance and glare are also still visible.

Mean centring

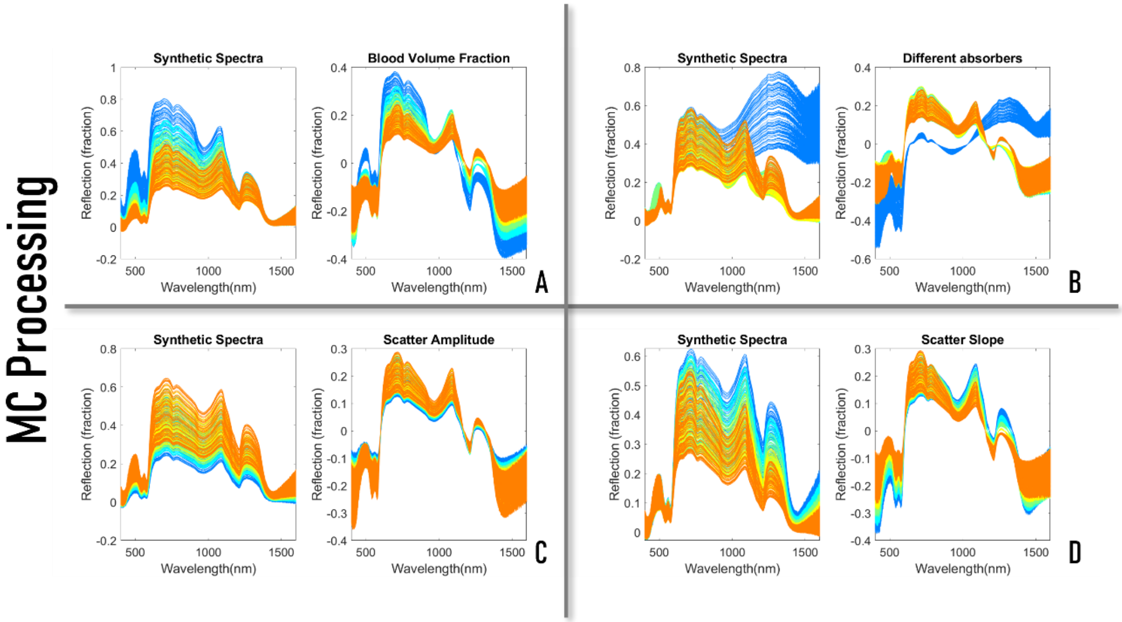

Figure S.4, Four different situations of changing parameters and the effect of MC on the spectra. The four spectra compared are; blood volume fraction(*A*), where it can be seen that a significant amount of overlap remains for the spectra, especially after 600 nm; different absorbers (*B*), where it can be seen that significant overlap remains in the spectra especially compared to other pre-processing techniques; scatter amplitude (*C*), here very little contrast can be seen but there is little reduction in the variations due to glare and distance; scatter slope (*D*), again some contrast is visible but the variations have not reduced significantly compared to other pre-processing techniques.

AUC

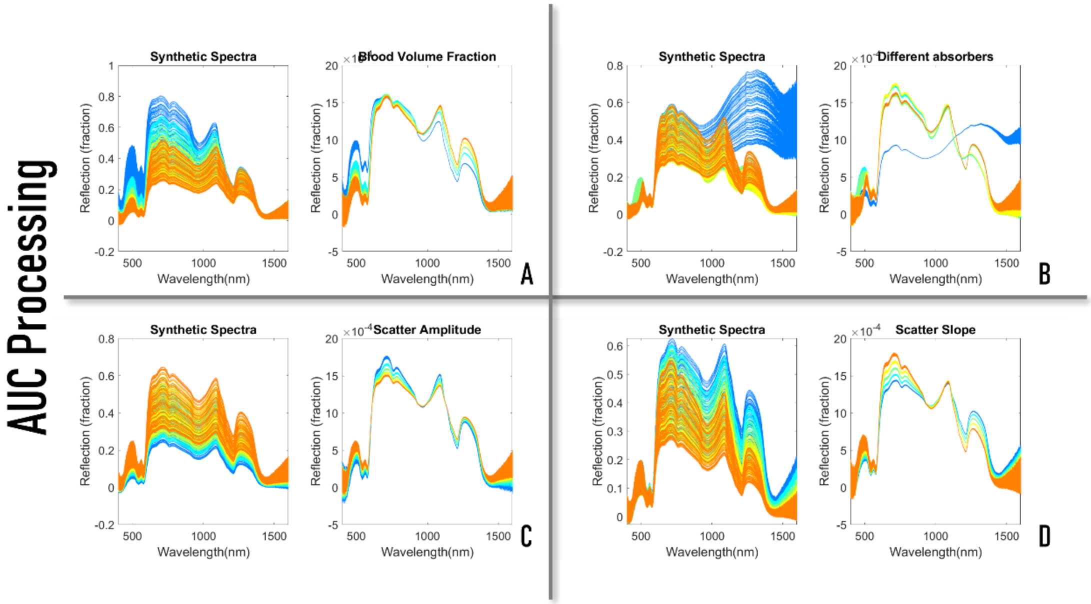

Figure S.5, Four different situations of changing parameters and the effect of AUC on the spectra. The four spectra compared are; blood volume fraction (*A*), most of the variations are removed from the spectra but the contrast is less visible especially in the start of the spectra; different absorbers (*B*), almost all the variations is removed from the spectra but the contrast in some places is not as distinct as SNV or MSC; scatter amplitude (*C*), most of the variations due to glare and distance are removed and there is contrast between the spectra; scatter slope (*D*), the variations in the spectra are reduced but less than the scatter amplitude, however there is clear contrast between the spectra.

Single wavelength

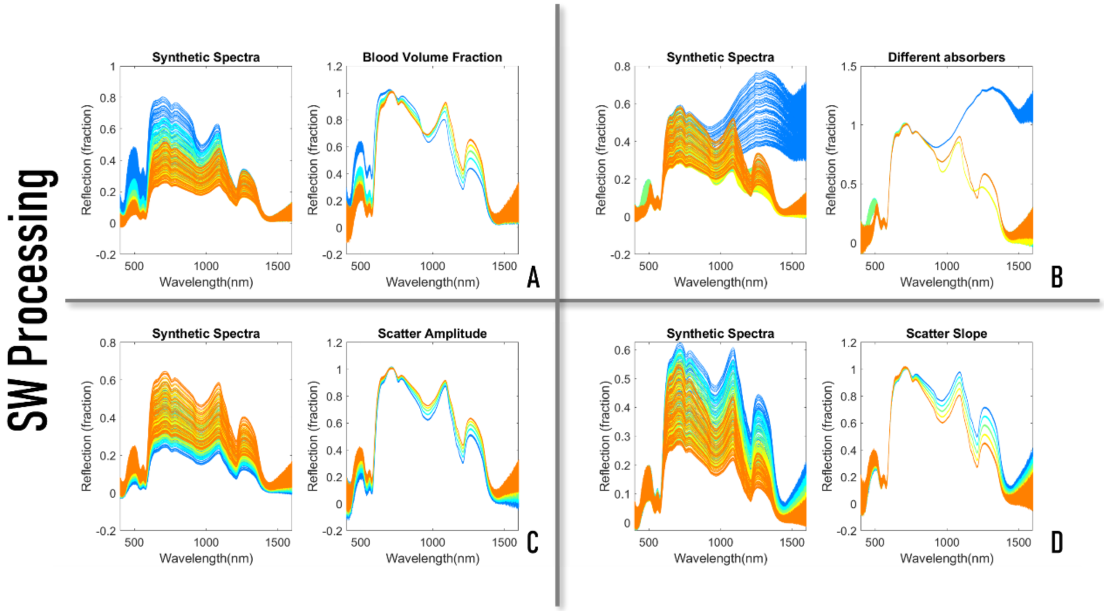

Figure S.6, Four different situations of changing parameters and the effect of SW on the spectra. The four spectra compared are; blood volume fraction (*A*), a large amount of the variations are removed but a some amount of the contrast is also removed; different absorbers (*B*), most of the variations due to the glare and distance are removed but a lot of the contrast to the blue spectra are removed; scatter amplitude (*C*), most of the variations due to glare and distance are removed and there is clear contrast between the spectra; scatter slope (*D*), the variations in the spectra are reduced at lower wavelengths but variations remain at the higher wavelengths.

First derivative

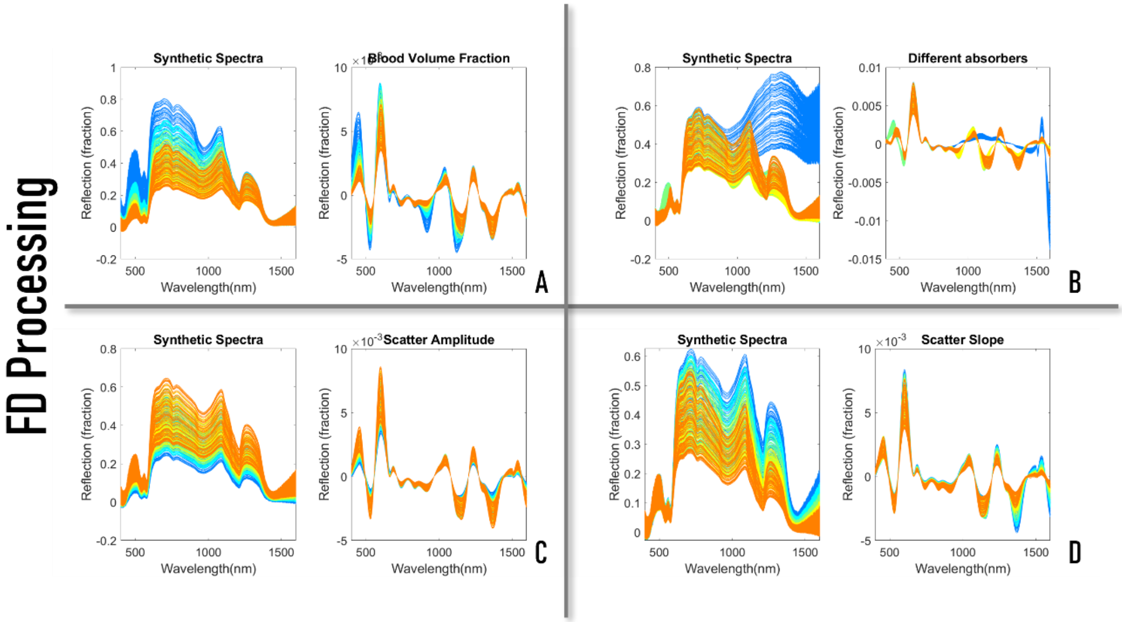

Figure S.7, Four different situations of changing parameters and the effect of FD on the spectra. Different from the other pre-processing techniques, FD changes the shape of the spectra significantly. The four spectra compared are; blood volume fraction (*A*), some variations due to the distance and glare remain in the spectra and the peaks are less clear compared to the spectra with different absorbers; different absorbers (*B*), the variations due to glare and distance are reduced and contrast can be seen between the spectra, however large parts of the spectra still overlap; scatter amplitude (*C*), similar to the BVF the variations are greatly reduced but the contrast between the spectra is difficult to see; scatter slope (*D*), the variations in the spectra are reduced and little contrast between the spectra are visible in the graph until above 1200 nm.

Second derivative

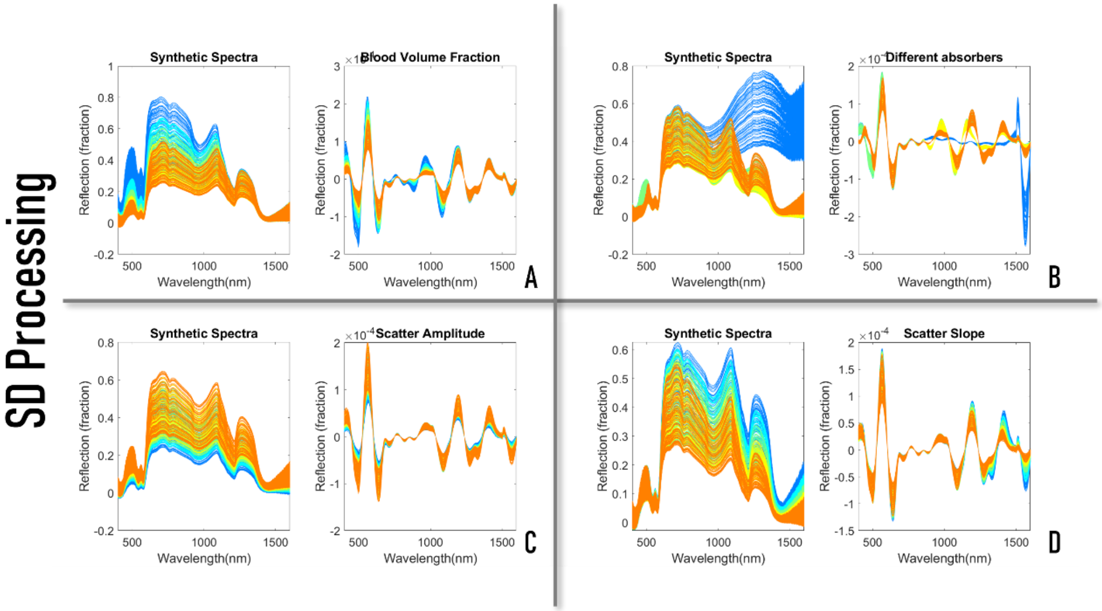

Figure S.8, Four different situations of changing parameters and the effect of SD on the spectra. Second derivative processing has the same problem as FD processing as it changes the shape of the spectra significantly, making the results more difficult to compare and interpret. The four spectra compared are; blood volume fraction (*A*), some variations due to the distance and glare remain in the spectra and the peaks are less clear compared to the spectra with different absorbers; different absorbers (*B*), the variations due to glare and distance are reduced and clear peaks can be seen between the spectra; scatter amplitude (*C*), similar to the BVF the variations are reduced but the contrast between the spectra is difficult to see; scatter slope (*D*), the variations in the spectra are reduced but the spectra are difficult to distinguish from each other although easier than for FD.
